# Spatial Distribution of Astins in *Aster tataricus* and Their Production by *Cyanodermella asteris*

**DOI:** 10.1021/acs.jnatprod.5c01383

**Published:** 2026-01-09

**Authors:** Diana A. Barrera-Adame, Taylor Priest, Timo H. J. Niedermeyer

**Affiliations:** † Department of Pharmaceutical Biology, Institute of Pharmacy, Freie Universität Berlin, 14195 Berlin, Germany; ‡ Department of Pharmaceutical Biology/Pharmacognosy, Institute of Pharmacy, Martin Luther University Halle-Wittenberg, 06120 Halle (Saale), Germany; § Max Planck Institute for Marine Microbiology, 28359 Bremen, Germany

## Abstract

*Aster tataricus* is a plant used in Traditional Chinese Medicine for the treatment of cough, phlegm, and asthma. Phytochemical studies of *A. tataricus* have resulted in the isolation of 23 peptides, among which the astins are recognized for their potential application as anticancer drugs. However, it was found that some of the astins, namely, astins C, F and G, are in fact produced by an endophytic fungus, *Cyanodermella asteris*, isolated from the inflorescences of the plant, while the remainder were suggested to be transformation products of these astins by *A. tataricus*. Using mass spectrometry imaging and microscopy, we demonstrate that astins exhibit a nonhomogeneous distribution, vary in relative abundance in different plant tissue sections, and are likely colocalized with fungal cells. To gain further insights into the diversity and composition of astins produced by *C. asteris*, we applied HPLC–MS/MS and mass spectrometry-based molecular networking after *in vitro* cultivation of the fungus in media with increased salinity. We found that the fungus produced a higher variety of astins than previously known, including several yet undescribed astins, suggesting that the fungus alone is indeed able to produce the complete astin diversity and that cross-species biosynthesis is unlikely.

## Graphical abstract

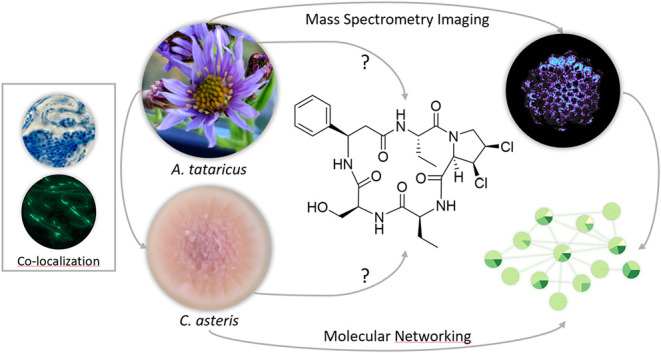

The plant *Aster tataricus* has been utilized in Traditional Chinese Medicine for more than two thousand years. Its dried roots and rhizomes are typically administered orally as decoctions to alleviate cough/asthma and eliminate phlegm.^1,2^ Numerous compounds, including terpenes, organic acids, flavonoids, and peptides, have been isolated from the drug. In addition, a total of 23 peptides have been described from the plant, called astins, asterinins, and tataricins.^2^

Astins (**1**–**9**) have been studied for their anticancer, immunosuppressive, and antioxidant activity.^3,4^ The best studied astin is astin C (**3**), a cyclic pentapeptide consisting of two l-2-aminobutyrates, one R-β-phenylalanine, one l-serine, and one l-*cis*-3,4-dichloroproline.^5,6^ 
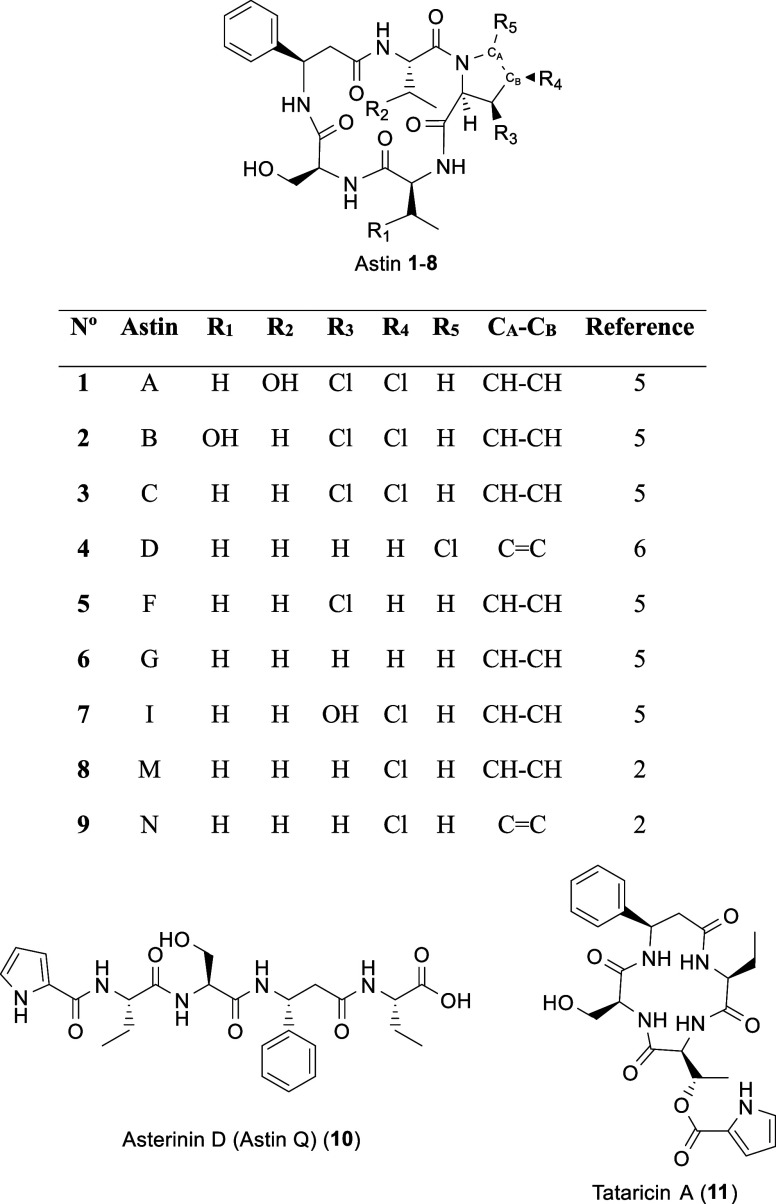


Although astins were originally thought to be produced by *A. tataricus*, the recent isolation and cultivation of a fungal endophyte changed this perspective. It was demonstrated that the fungal endophyte, *Cyanodermella asteris*,^7^ was capable of producing astin C (**3**), astin F (**5**), and astin G (**6**), but not the other astins observed *in planta*.^8,9^ Therefore, it was hypothesized that the fungal endophyte produces some astins, but the wider diversity of astins in the plant was a result of the plant metabolizing the fungal natural products (cross-species metabolic pathway).^8^

In recent years, the specialized metabolite distribution in plant tissues has been explored in more detail, facilitated by the improvement of mass spectrometry imaging instruments, with matrix-assisted laser desorption ionization mass spectrometry imaging (MALDI-MSI) being used most often.^10–13^ This technique can resolve metabolite distribution and can also support the detection of metabolite structural diversity in biological tissues by molecular networking techniques,^14^ making it a powerful tool for the visualization of metabolite spatial distribution via the generation of ion images of detected analytes. The ionization of analytes is performed directly on the surface of the sample, thereby eliminating the necessity for extraction.^15^ A technique employed to study the spatial localization of microorganisms is fluorescence *in situ* hybridization (FISH), allowing the *in situ* visualization of microbial populations in their natural environments.^16^ This technique employs fluorescent rRNA-targeted oligonucleotide probes that bind to a specific sequence of rRNA in intact ribosomes.^17^ The combination of MSI and FISH has, for example, been used to study the relationship of host–microbe symbioses of *Bathymodiolus*, a deep-sea mussel, and its endosymbiotic bacteria.^18^ Imaging and identification of metabolites and in parallel visualizing the distribution of microorganisms in a host at high resolution are highly effective workflows to study organismic interactions. However, *in planta*, the analysis of potential relationships between, e.g., an endophyte fungus, the host plant, and their specialized metabolism is still challenging due to the potentially low-signal intensity of compounds in MALDI-MSI experiments and competition between the innate autofluorescence of plant tissues and the FISH probe.^19^

In this study, we wanted to explore whether MALDI-MSI could be used to study the distribution of astins *in planta* and to elucidate the contribution of the fungus and the plant to astin diversity by localizing the astin-producing endophyte in the plant using FISH. We observed a nonhomogeneous distribution of astins in the plant tissues in areas where we could visualize fungal cells using classical microscopy staining techniques, suggesting that the astins were produced by the fungus in spatially confined environments within the plant. FISH analyses proved to be difficult due to plant tissue autofluorescence. Astin production by *C. asteris* was reevaluated by HPLC–MS and mass spectrometry-based molecular networking. We found that increased medium salinity resulted in the production of many known and several yet undescribed astins, suggesting that the fungus alone is able to produce the known astins.

## Results and Discussion

### Astin Diversity and Distribution in Plant Tissues

The diversity and distribution of astins and related peptides in various *A. tataricus* tissues were studied by HPLC–MS in combination with classical molecular networking using global natural products social (GNPS) molecular networking as well as MALDI-MSI. These methods allowed us to compare and evaluate the presence, relative abundance, and localization of the peptides in the tissues, as shown in Figure 1.

**Figure 1:**
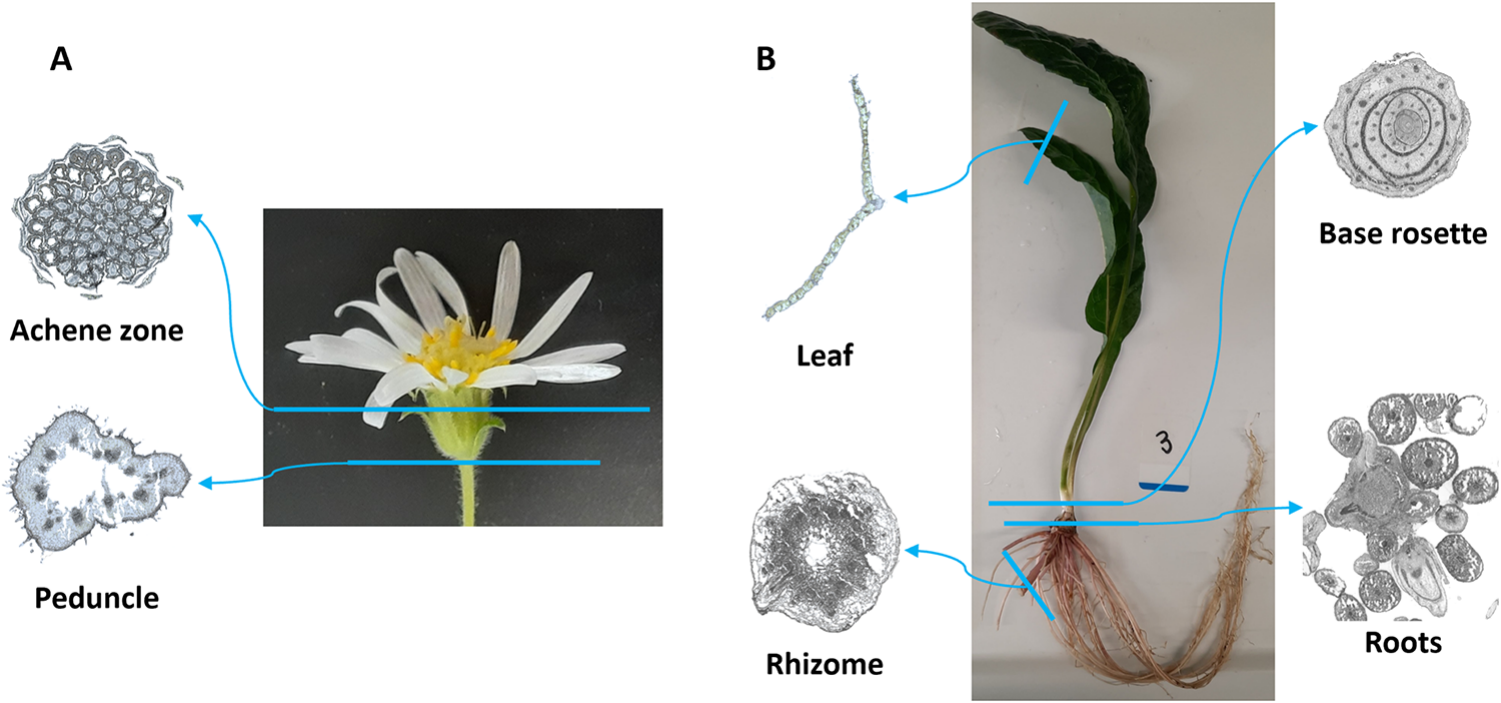
Tissues and transversal sections of *A. tataricus* (A) flower and (B) plant body analyzed in this study.

GNPS analysis of the HPLC–MS data (Figure 2) showed a network of higher-abundant compounds composed of 6 astins and asterinin D (**10**), a linear astin derivative considered an artifact.^20^ The second isolated node corresponded to tataricin A (**11**), an astin-related cyclic tetrapeptide. Within the network, the two predominant nodes corresponded to astins A/B (**1**/**2**) and C (**3**), suggesting that they were the most abundant astins in the analyzed tissues. **1** and **2** are isomeric compounds, but due to their structural differences, they can be distinguished by tandem mass spectrometry. As a fragment ion at *m*/*z* 338.0 observed in the main astin peak in our HPLC–MS analyses (Figure S1b and c), which has been reported for **1**, but not for **2**,^8^ **1** was present in the studied tissues. However, the peak in the chromatogram also showed a small shoulder (with the same *m*/*z*), which could indicate the presence of **2** in smaller quantities (Figure S1).

**Figure 2:**
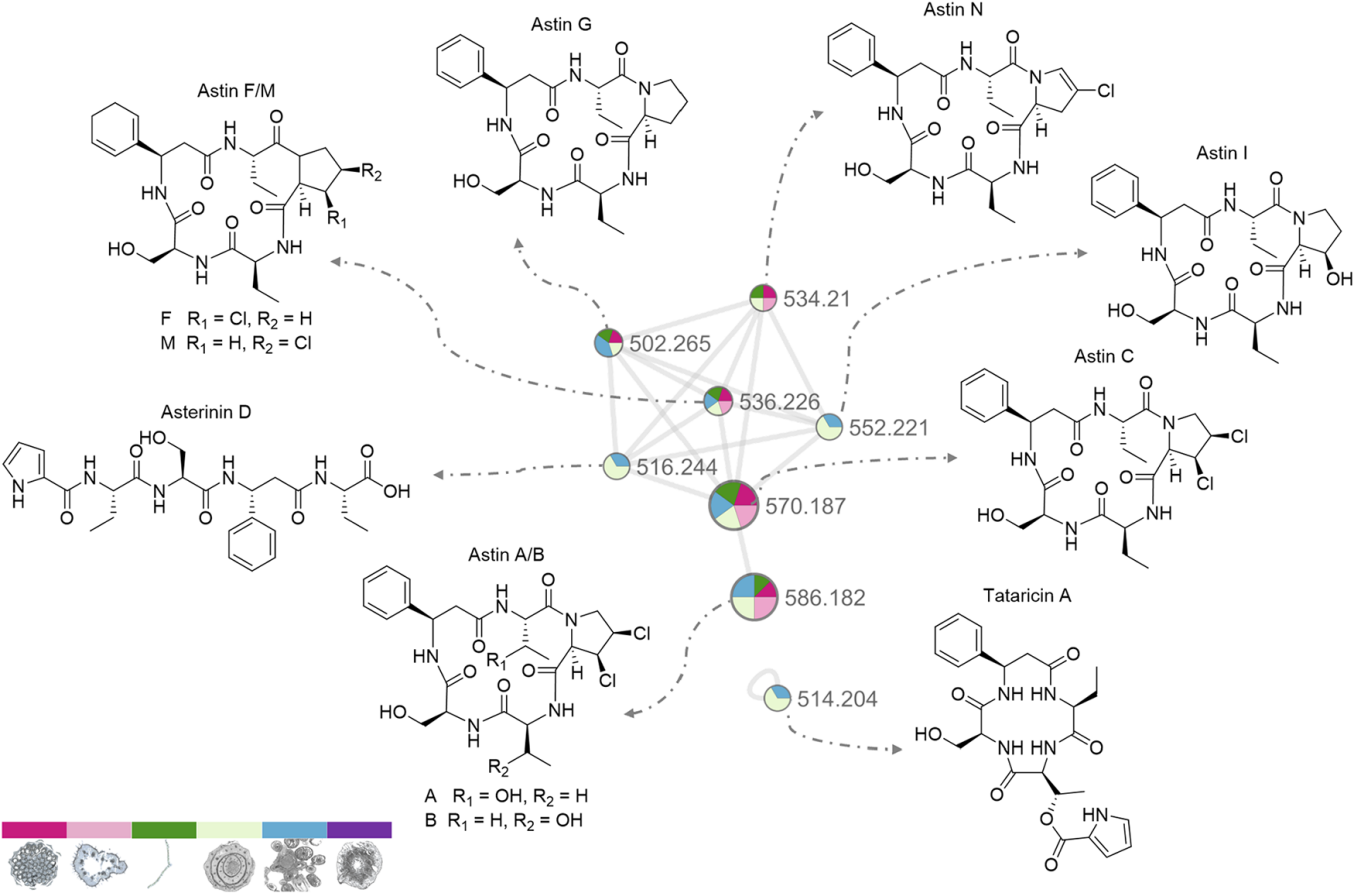
GNPS analysis of astins and related compounds in *A. tataricus* tissues. Dark pink: achene zone; light pink: peduncle; dark green: leaf; light green: base rosette; blue: root; purple: rhizome. Node and section sizes are based on precursor intensity. Compound annotation is based on the available reference material (astin C), MS/MS fragmentation (astins A and B), and accurate mass of known astins.

**1**/**2**, **3**, and astins F/M (**5**/**8**) were present in all of the tissues except in the rhizome, a tissue in which none of the astins could be detected. **6** was not detected in the peduncle, while astin I (**7**) and **10** could only be detected in the base rosette and roots. The astins known to be produced by the fungus, **3**, **5**, and **6**, were all detected in the plant.

The chemical compositions of the basal rosette and roots were similar, which may be due to their spatial proximity. Remarkably, these tissues contained **7**, **10**, and **11**, compounds that were not detected in other parts of the plant. In contrast, astins D/N (**4**/**9**) were present only in the aerial part of the plant. Both could indicate that the biosynthesis of specific astin derivatives was favored by the respective conditions in the microenvironments in the subterranean or aerial organs of the plant.

The HPLC–MS analyses results were, in general, confirmed by evaluation of the MSI data (Figure 3, Tables S1 and S2). Comparing the abundance of astins, we observed that compounds **1**/**2** and **3** exhibited high signal intensity in both experiments. Interestingly, the signal intensity of **10** was much higher in MSI compared to HPLC–MS, which might be due to the different ionization methods (ESI vs MALDI). **10** as well as **1**/**2**, **3**, **5**/**8**, and **6** were found with high relative intensity in the achene zone, the roots, and the base rosette. These tissues stand out for having the highest signal intensities, a high diversity of astins, and a broader distribution over the tissue. In turn, **11** also showed high-ion intensity, although it was limited to the lower tissues of the plant. Using MALDI-MSI, **4**/**9** and **7** were barely detected. Interestingly, we could detect only minimal amounts of astins in the leaf tissue. Again, no astins could be detected in the rhizome (Figure S2).

**Figure 3:**
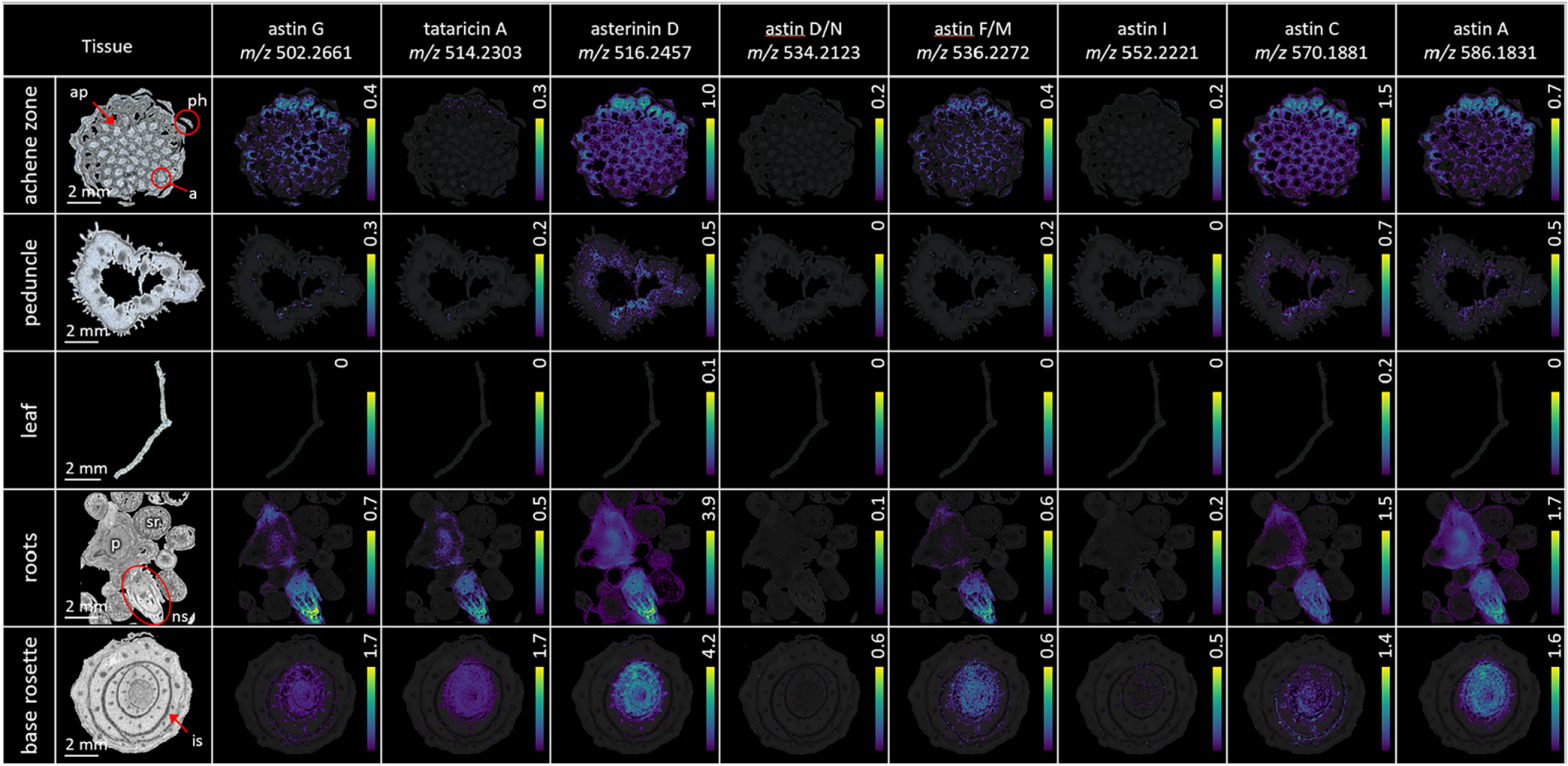
Distribution and normalized relative abundance of astins and related compounds in tissues of the plant *A. tataricus*. Longitudinal tissue sections (thickness: 14 μm) of the achene zone with achene (a), achene pericarp (ap), phyllaries (ph), the peduncle, the leaf, the roots with pith (p), new shoots (ns), secondary root (sr), and the base rosette with interleaf space (is). MALDI-MSI in the pos. mode, relative ion distributions are displayed as heat maps (black for 0 ion detected, yellow for the maximum percentage of ion intensity; percentages adjusted by compound ion intensity); images normalized to the total ion intensity.

In general, the distribution and intensity of astin signals in specific tissues were neither specific nor homogeneous: even though some regions of the plant had a tendency to contain astins at concentrations higher than those of other parts, the distribution of the compounds in the respective tissue did not show a strict distribution. We were particularly intrigued by the observed astin distribution in the base rosette and achene zone: the ions were predominantly detected within the spaces outside the plant tissues, the interleaf space in the base rosette, and the space between the achenes in the flower. For this reason, we decided to study the base rosette in more detail in a microscopic study.

### Microscopic Analysis of the Base Rosette

In Figure 4, micrographs of the basal rosette are shown. Between the two leaves, an interleaf space was observed (Figure 4a). Interestingly, in some parts of the basal rosette, this interleaf space looked as if it contained cell debris or other unidentified granular structures (Figure 4b). Although these granular structures were present in a large part of the interleaf space, their distribution was not homogeneous, with some spaces between the patches of these structures.

**Figure 4:**
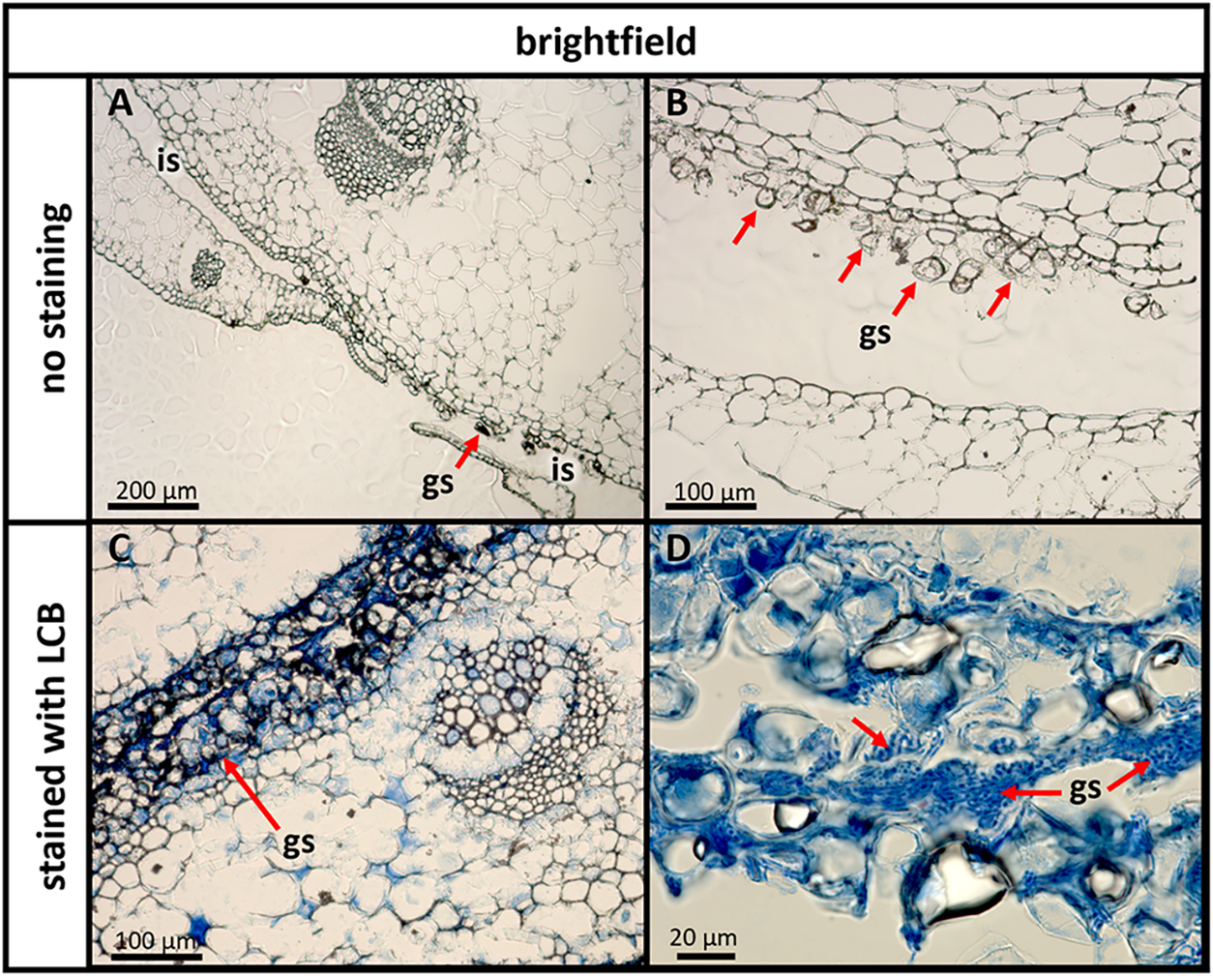
Bright-field micrographs showing transversal cuts of an *A. tataricus* basal rosette. (A) Interleaf space (is) without (upper interleaf space) and with granular structures (gs). (B) Magnified image of granular structures (gs) between leaves. (C) Tissue treated with lactophenol cotton blue with stained granular structures (gs). (D) Higher magnification image of interleaf space (is) and granular structures (gs) stained with lactophenol cotton blue.

To characterize these granular structures that were present in the interleaf space, the tissue was stained with lactophenol cotton blue (LCB). This dye has been widely used in the identification of fungi, since it reacts with the chitin present in fungal cell walls.^21,22^ As can be seen in the micrographs (Figure 4c and d), the structures were stained, suggesting that they were composed of fungal cells. Given the colocalization with the astins, we hypothesized that this fungus was *C. asteris*. To test this hypothesis, we established a method for the specific and direct detection of the fungus in the tissue by CARD-FISH. However, we found the autofluorescence of the plant tissues to be an unsurmountable obstacle that in our hands made it impossible to visualize the localization of *C. asteris* by FISH *in planta* (for further details, see the Supporting Information).

### *In Vitro* *C. asteris* Cultivation under Increased Salinity

Many astins contain one or more chlorine atoms. Production of halogenated metabolites can be enhanced when salts like NaCl were added to the culture medium.^23^ Previous experiments indicated that **3**, **5**, and **6** were produced by *C. asteris* when grown in peptone-containing medium (less than 1% NaCl).^8,24^ Thus, we modified the salinity of the cultivation medium and cultivated *C. asteris* in media supplemented with NaCl. Samples were analyzed by HPLC–MS, followed by mass spectrometry-based molecular networking analysis and identification of the astin derivatives based on HRMS and tandem-MS data (Figure S3).

As shown in Figure 5, we could confirm that in standard MEA medium, **3**, **5**/**8**, and **6** were produced, and we could also detect the production of **10** in this medium. No other astins than the ones already described from *C. asteris* were detected when the strain was cultivated in the MEA medium.

**Figure 5:**
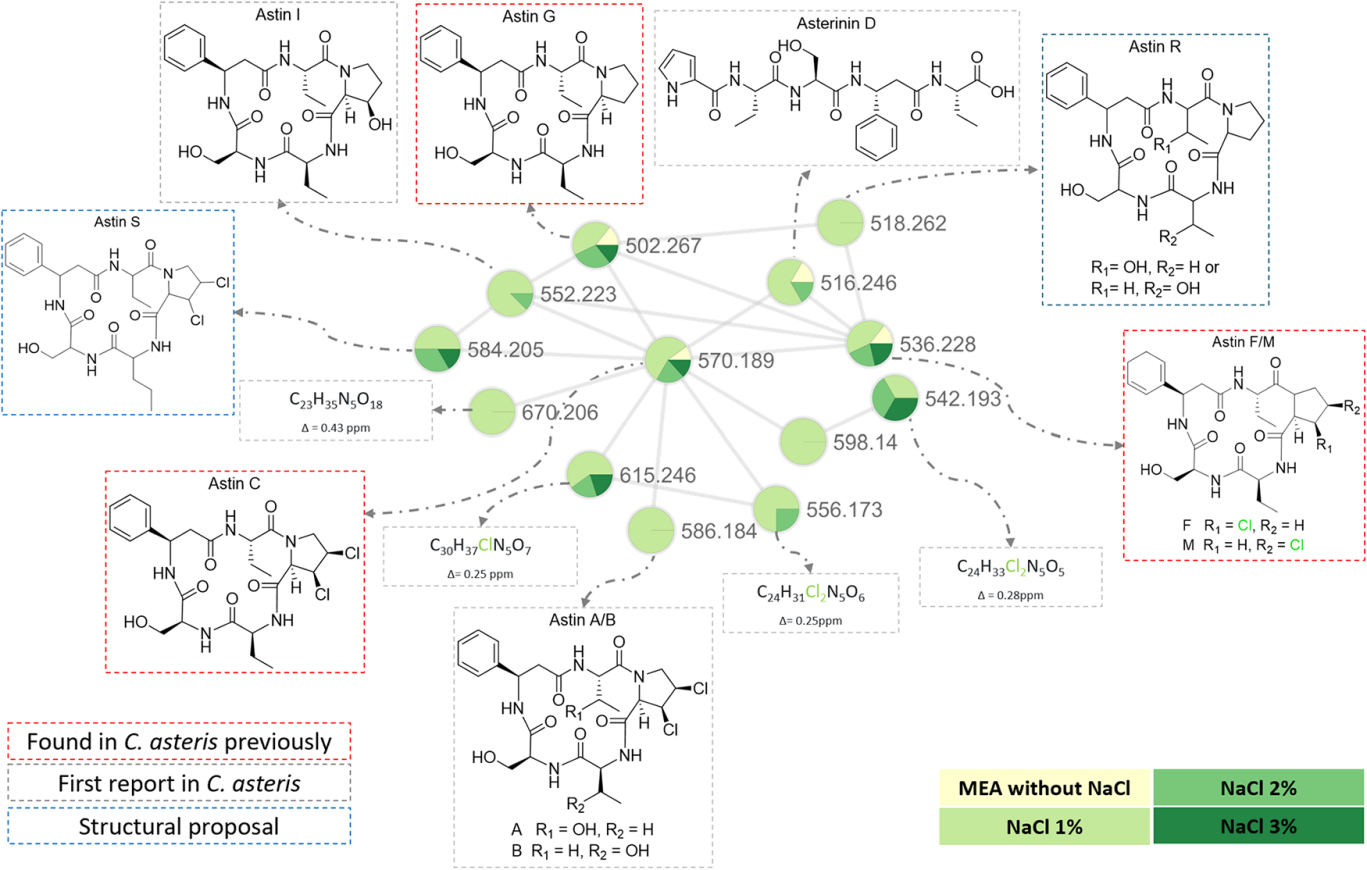
GNPS molecular network of ions corresponding to astin derivatives produced by *C. asteris* cultivated in a NaCl-supplemented medium. The sum formula of the analyte with *m*/*z* 598.14 could not be unambiguously determined.

We observed that the production of these compounds increased when the concentration of NaCl in the medium was increased by 1%. Also, at this concentration, the fungus started producing other astins that had formerly only been described from the plant: **1**/**2** and **7** were not detected in the nonsupplemented medium but found in the medium supplemented with 1% of NaCl. This indicates that the fungus is capable of producing the observed astin diversity without requiring cross-species biosynthesis pathways (such as the previously proposed hydroxylation of fungus-produced **3** by the plant to yield **1**/**2**).^8^ Rather, the biosynthesis of the astins seems to depend on the environment in which the fungus grows in. Furthermore, putative astin derivatives that have not been previously reported from *A. tataricus* or *C. asteris* were detected in the media with increased salinity. For two of them, we were able to propose structures based on the scaffold and structural modifications of known astins as well as on the MS/MS fragmentation patterns (Figure S4): the compound detected at *m*/*z* 518.2620, C_25_H_35_N_5_O_7_ (Δ 2.1 ppm), is the second nonhalogenated astin reported to date, for which we have suggested the putative structure shown in Figure 5 (astin R). For the compound detected at *m*/*z* 584.2048, C_26_H_35_Cl_2_N_5_O_6_ (Δ 1.9 ppm), we were also able to suggest a structure (astin S). The astin GNPS network contained additional nodes; however, for these compounds, structure prediction was less straightforward.

An increase of the medium salinity by 2 or 3% did not result in the production of additional astins or increased yields, indicating that the addition of 1% NaCl is sufficient to trigger astin production. Usually, plants and fungi are affected by the presence of salt in natural environments. The usual concentrations of salt in soil are classified as nonsaline (0–0.3%), slightly saline (0.3–0.6%), medium saline (0.6–1.2%), and highly saline (more than 1.2%).^25^ No significant effect on *C. asteris* growth was observed under medium saline concentrations (1% NaCl), while at 2 and 3%, a decrease in the number and diameter of the germinated colonies was observed.

In conclusion, mass spectrometry imaging and microscopic studies suggest that the fungus *C. asteris* is the sole producer of astins in the plant *A. tataricus*, as the spatial distribution of astins in the plant tissues was nonhomogeneous, astins were colocalized with each other, and they were localized mainly outside of the plant tissues. Indeed, cultivation of *C. asteris* in a medium with increased salinity showed that the astin structural diversity increased when the NaCl concentration increased by 1%.

## Experimental Section

### General Experimental Procedures

Extracts were analyzed by HPLC–HR-ESI-MS^2^ using a Q Exactive Plus mass spectrometer (Thermo Fisher Scientific), equipped with a heated electrospray ionization (ESI) interface, coupled to an UltiMate 3000 HPLC system (Thermo Fisher Scientific). Ionization was performed in positive and negative ionization modes. The capillary temperature was kept at 350 °C. Mass range: *m*/*z* 450 to 750 for extracts from *C. asteris* in NaCl-supplemented medium; *m*/*z* 100 to 1000 for extracts from *A. tataricus* tissues. Chromatography was performed on a Kinetex C18 column (50 × 2.1 mm, 2.6 μm, 100 Å) (Phenomenex), eluted with H_2_O (A) and MeCN (B) (0.1% formic acid each), using the following gradient: 5% to 100% A (0–16 min), 100% B (16–20 min), and flow rate (0.4 mL/min). Astin C was available as isolated standard (provided by Dr. Schafhauser, University of Tübingen).

### Samples

*Cyanodermella asteris* (Mycobank accession number #814158) was provided by Dr. T. Schafhauser and Prof. Dr. W. Wohlleben (University of Tübingen, Germany) in August 2019. *Aster tataricus* plants were bought from SARASTRO-STAUDEN, Austria, and cultivated in a private garden in Halle (Saale), Germany, under natural conditions in October 2021.

### Mass Spectrometry Imaging

MSI Sample Preparation: Achene zone, peduncle, leaf, root, base rosette, and rhizome from *A. tataricus* were harvested, embedded in a gelatin solution (10%, w/v), and immediately frozen in liquid nitrogen to form a solid block. Embedded samples were stored at −70 °C until sectioning. The tissues were sectioned with a thickness of 14 μm at −21 °C using a cryotome (MICROM HM 500 M, MICROM International GmbH), thaw-mounted on VWR Superfrost Plus slides, and dried in a desiccator over silica gel for 1 h. The samples were first observed using an inverse microscope (Axio Observer, Zeiss), and images were taken with an Axiocam 712 color digital camera for later comparison with the MSI results. To perform HPLC–MS analysis of plant tissues, some sections were collected in tubes and later extracted with MeOH (5 mg tissue per mL solvent).

### MALDI-MSI Analyses

Atmospheric pressure MALDI-MSI measurements were performed on a Fourier transform orbital trapping mass spectrometer (Q Exactive Plus, Thermo Fisher Scientific) equipped with an AP-MALDI (ng) UHR source (MassTech) with a laser spot size <10 μm. Imaging experiments were conducted in positive ion mode in the scanning range *m*/*z* 100–1000, with 140,000 resolution at *m*/*z* 200, one microscan, 5 × 10^6^ AGC target, 500 ms maximum injection time, 4.5 kV spray voltage, 450 °C capillary temperature, and 60% for the S-lens RF value. The MALDI source parameters were adjusted as follows: CSR mode (constant speed rastering), scanning velocity of 2.30 mm/min for 20 μm and 3.45 mm/min for 30 μm pixel size, 6 kHz pulse rate, 31% laser energy. The centroid raw data were converted from the Thermo .raw files to .imzML using MassTech imzMLConverter (ng) 1.0.1 (merge strategy “Average”) and normalized by TIC. The converted files were analyzed with MSi Reader (v 1.01). All images were linearly interpolated in order 3, with *m*/*z* ± 5 ppm tolerance. For SMART parameters,^26^ see Table S1.

### Microscopy Analysis of *A. tataricus* Tissues

A base rosette tissue was cut in the microtome as described before with a thickness of 2 mm, stained for 5 min with lactophenol cotton blue (LCB) solution (Sigma-Aldrich), and microphotographed using an inverse microscope (Axio Observer, Zeiss), equipped with 10, 20, and 63× objective lenses, an Axiocam 712 color digital camera, and fluorescence filter cubes for Alexa 488 (emission: 525 nm; excitation: 490 nm) and DAPI (emission: 470 nm; excitation: 350 nm).

### *In Vitro* Cultivation of *C. asteris* under Increased Salinity

*C. asteris* was cultivated on malt extract agar (MEA) medium (12.8 g maltose, 2.8 g dextrin, 2.4 g glycerol, 0.8 g gelatin peptone, and 15 g/L agar) plates, supplemented with NaCl (0, 1, 2, or 3%). A 5 mm diameter agar plug with the fungus was placed on each media plate. The plates were incubated at 24 °C and 16 h of light (intensity: 105 μmol/m^2^/s) per day for 21 days in total. After cultivation, the medium and mycelium were homogenized with an Ultra-Turrax. The homogenate was extracted 4 times with 400 mL of EtOAc, stirring at 175 rpm. The solvent was removed using a rotary evaporator and redissolved in MeOH to 1 mg/mL for subsequent HPLC–MS analysis.

### Classical Molecular Networking

Raw mass spectrometry data files were converted from .RAW to .mzML format using MSConvert from ProteoWizard (version 3.0.24002).^27^ A scan polarity filter was used during data conversion to separate positive ion mode scans, facilitating a more targeted analysis in the subsequent data analysis steps. The molecular networks for CMN analysis were generated using GNPS.^28^ The precursor ion mass tolerance was set to 0.02 Da, and the same 0.02 Da tolerance was applied to HRMS^2^ fragment ions. The network was constructed with edges retained if they exhibited a cosine score exceeding 0.7 and a library score threshold of 0.7. The minimum number of matched fragment ions was adjusted to 9 for *C. asteris* in NaCl-supplemented media and 6 for *A. tataricus* tissue extracts. Additionally, an MEA extract file was used as a blank for *C. asteris* in the NaCl-supplemented media experiment. Molecular networks generated by the GNPS workflow were visualized and analyzed using Cytoscape (version 3.9.0).^29^

## Supplementary Material



## Notes

HPLC–MS chromatograms of *A. tataricus* tissues, SMART parameters of the MSI experiments, MSI images of *the A. tataricus* rhizome, MS and MS/MS spectra of the analyzed astins, and method and results of FISH experiments (PDF)

## ∥

Bioanalytical Sciences and Food Analysis, University of Bayreuth, 95447 Bayreuth, Germany

## ⊥

Institute of Microbiology, Department of Biology, ETH Zurich, 8092 Zurich, Switzerland.
