# Supplementary material for: Spatial Distribution of Astins in Aster tataricus and Their Production by Cyanodermella asteris
Source: J Nat Prod. 2026 Jan 9;89(1):259–66. doi: 10.1021/acs.jnatprod.5c01383 (PMC12836360; doi:10.1021/acs.jnatprod.5c01383)
Supplement: Supplementary file 1 [file np5c01383_si_001.pdf]

Supporting Information

Spatial distribution of astins in Aster tataricus and their production by Cyanodermella asteris

Diana A. Barrera-Adame[†,‡], *Taylor Priest*[§,‖], *Timo H. J. Niedermeyer*[†,*].

[†]Department of Pharmaceutical Biology, Institute of Pharmacy, Freie Universität Berlin, 14195 Berlin, Germany; part of this work was conducted at the Department of Pharmaceutical Biology/Pharmacognosy, Institute of Pharmacy, Martin Luther University Halle-Wittenberg, 06120 Halle (Saale), Germany

[§]Max Planck Institute for Marine Microbiology, 28359 Bremen, Germany

[‡]Current affiliation: Bioanalytical Sciences and Food Analysis, University of Bayreuth, 95447, Bayreuth, Germany

[‖]Current affiliation: Centre of Origin and Prevalence of Life, ETH Zurich, 8092 Zurich, Switzerland

[*]corresponding author, timo.niedermeyer@fu-berlin.de; +49 30 83875060

Figure S1. A. HPLC-MS Chromatograms of *A. tataricus* tissues (pos. mode). B. Extracted Ion Chromatograms (EIC) of m/z 586.1823 (± 5 ppm). C. Characteristic MS^2-fragments of astin A and B. D. MS/MS data for Astin A/B in root tissue of *A. tataricus*, showing the main compound to be Astin A. 3

Figure S2. Ion image of astins in A. tataricus rhizome tissue. 6

Figure S3. MS, MS/MS, and GNPS nodes for compounds detected in *in vitro* cultivation of *C. asteris* under increased salinity (Astins A/B, C, F/M, G, I, R, S, Asterinin D, as well as selected yet uncharacterized astins). 6

Figure S4. MS/MS data and main fragment interpretation of the postulated astins R and S. 10

Figure S5. Micrographs of *C. asteris* after CARD-FISH hybridization (C_ast_HRP probe, labeled with AlexaFluor448, DAPI-stained) with different concentrations of formamide. a, d. 10% formamide. b, d. 20% formamide. c, f. 30% formamide. Displayed in channel green (3000 ms) and blue (120 ms). Hyphae (h), spores (s), and nuclei (n). 13

Figure S6. Micrographs of *C. asteris* after CARD-FISH hybridization (C_ast_HRP probe, labeled with AlexaFluor448) with different concentrations of formaldehyde and at different temperatures. Displayed in channel green (3000 ms) 13

Figure S7. Micrographs of the transversal stem section from *A. tataricus* with treatments to reduce the autofluorescence on plant tissue (14 µm thickness). Tissues treated with agents to reduce autofluorescence. Epidermis (e), ground tissue system (g), and vascular tissue system (v). Displayed in brightfield and UV channels blue, green, and red. 15

Figure S8. CARD-FISH visualization of *C. asteris* on *A. tataricus* tissue. a-c. hybridization with C_ast_HRP probe labeled with AlexaFluor448 and DAPI stain. d-f. hybridization with NON_HRP probe labeled with AlexaFluor448 and DAPI stain. Displayed in channels, blue (150 ms) and green (3000 ms). *A. tataricus* epidermis (e) and putative *C. asteris* hyphae (h) and nucleus (n). 16

Figure S1. A. HPLC-MS chromatograms of *A. tataricus* tissues (pos. mode). B. Extracted Ion Chromatograms (EIC) of *m/z* 586.1823 (± 5 ppm). C. Characteristic MS/MS-fragments of astin A and B. D. MS/MS data for Astin A/B in root tissue of *A. tataricus*, showing the main compound to be Astin A.

A.

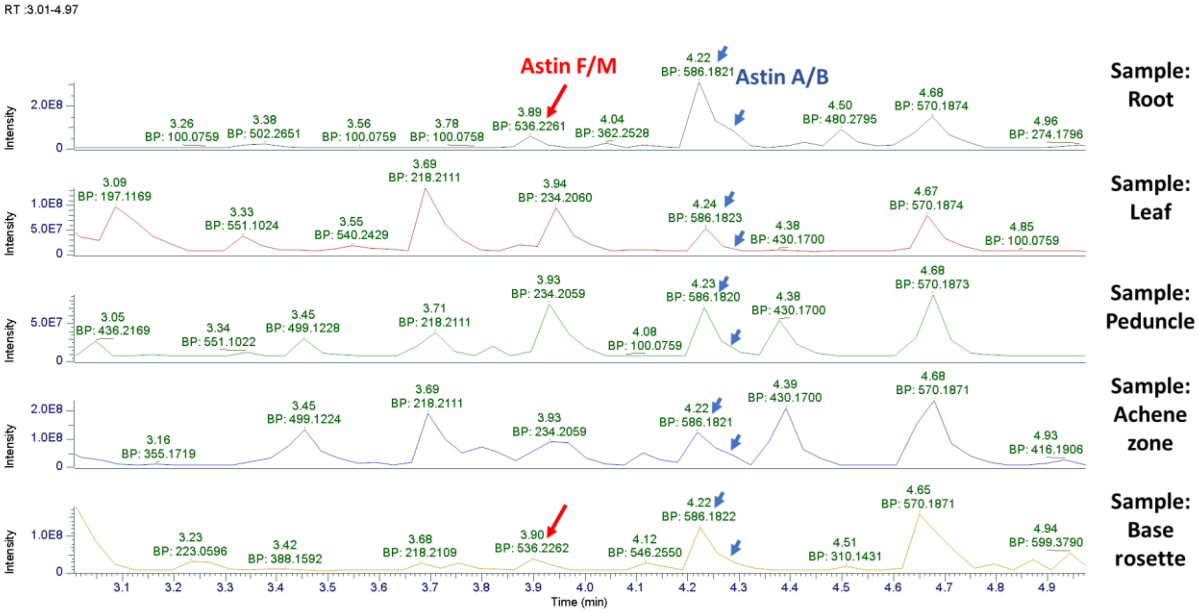

B.

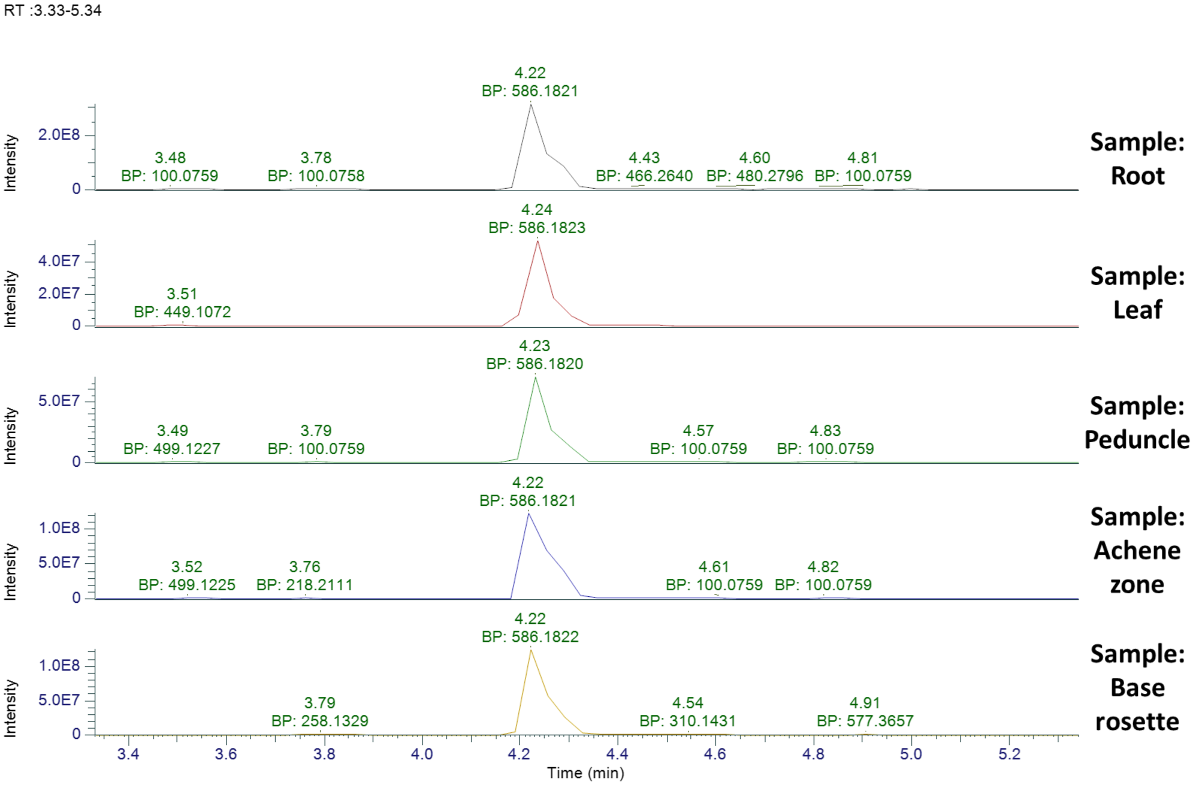

C.

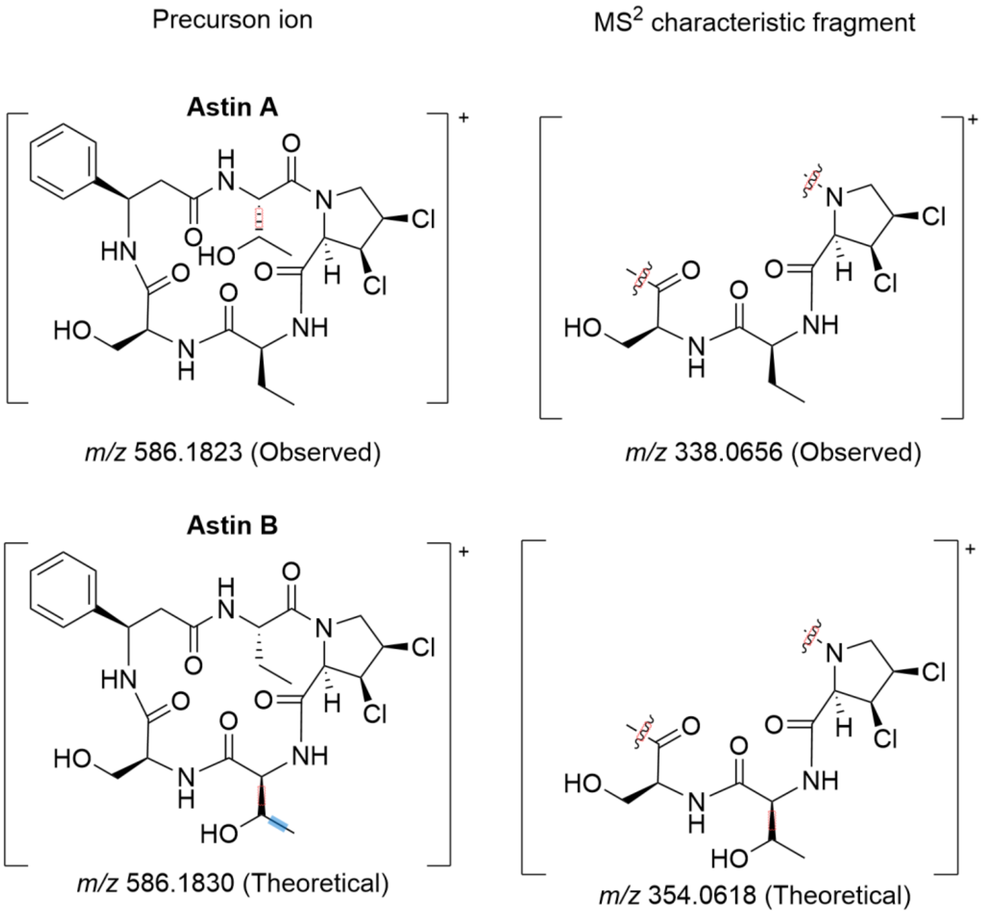
Precurson ion
MS2 characteristic fragment
Astin A
m/z 586.1823 (Observed)
m/z 338.0656 (Observed)
Astin B
m/z 586.1830 (Theoretical)
m/z 354.0618 (Theoretical)

D.

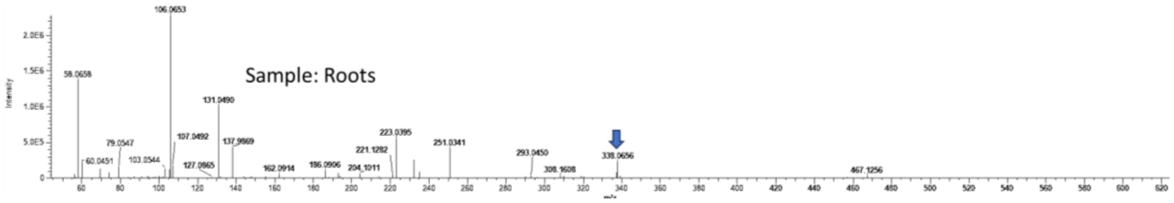
Sample: Roots
106.0653
58.0658
131.0490
223.0395
251.0341
338.0656
467.1256

Table S1. SMART parameters for the MS-Imaging analysis of *A. tataricus tissues.*

***A. tataricus* sample**	**SMART parameters**	
Achene zone	Step size	30 µm x 30 µm
	Total scans	74760
	Acquisition time (h)	11.51
Peduncle	Step size	20 µm x 20 µm
	Total scans	47000
	Acquisition time (h)	7.1
Leaf	Step size	20 µm x 20 µm
	Total scans	92000
	Acquisition time (h)	14
Base rosette	Step size	30 µm x 30 µm
	Total scans	78400
	Acquisition time (h)	12.1
Root	Step size	30 µm x 30 µm
	Total scans	122211
	Acquisition time (h)	18.8
Rhizome	Step size	20 µm x 20 µm
	Total scans	44100
	Acquisition time (h)	6.7

(**S**): Spot size of the MALDI source: <10 µm. Experiments conducted in Constant Speed Rastering mode, scanning velocity 2.28 mm/min for 20 µm, 3.42 mm/min for 30 µm pixel size. Total scans see table below. (**M**): Molecular identification is based on high accuracy masses as shown in Table S2 (**A**): The compounds listed in Table S2 have been studied. (**R**): Resolution set to 140,000 at m/z 200. (**T**): Acquisition time see table above.

Table S2. Accurate mass measurement of astin congeners in *A. tataricus* tissue using MALDI-MSI.

**Astin**	**Formula**	**Exact Mass**	**$[M + H]^+$**		
			**theoretical**	**observed**	**error (ppm)**
Astin A/B	$C_{25}H_{33}Cl_2N_5O_7$	585.1757	586.1830	586.1831	0.2
Astin C	$C_{25}H_{33}Cl_2N_5O_6$	569.1808	570.1881	570.1881	0.1
Astin D/N	$C_{25}H_{32}ClN_5O_6$	533.2041	534.2114	534.2123	1.7
Astin F/M	$C_{25}H_{34}ClN_5O_6$	535.2198	536.2270	536.2272	0.2
Astin G	$C_{25}H_{35}N_5O_6$	501.2587	502.2660	502.2661	0.1
Astin I	$C_{25}H_{34}ClN_5O_7$	551.2147	552.2220	552.2221	0.2
Astin Q (Asterinin D)	$C_{25}H_{33}N_5O_7$	515.2380	516.2453	516.2457	0.9
Astin R	$C_{25}H_{35}N_5O_7$	517.2537	518.2609	518.2620	2.1
Astin S	$C_{26}H_{35}Cl_2N_5O_6$	583.1964	584.2037	584.2048	1.9
Tataricin A	$C_{25}H_{31}N_5O_7$	513.2224	514.2296	514.2303	1.3

Figure S2. Ion image of astins in *A. tataricus* rhizome tissue.

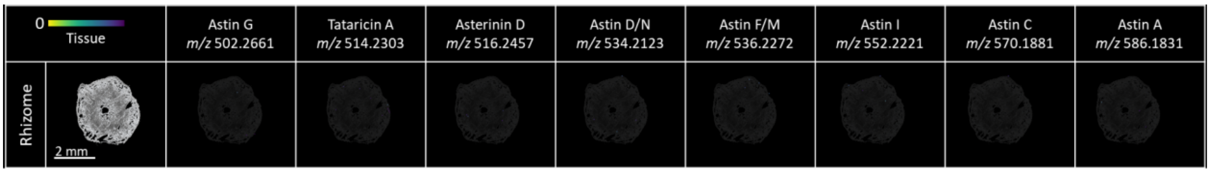

Figure S3. MS, MS/MS, and GNPS nodes for compounds detected in *in vitro* cultivation of *C. asteris* under increased salinity (Astins A/B, C, F/M, G, I, R, S, Asterinin D, as well as selected yet uncharacterized astins).

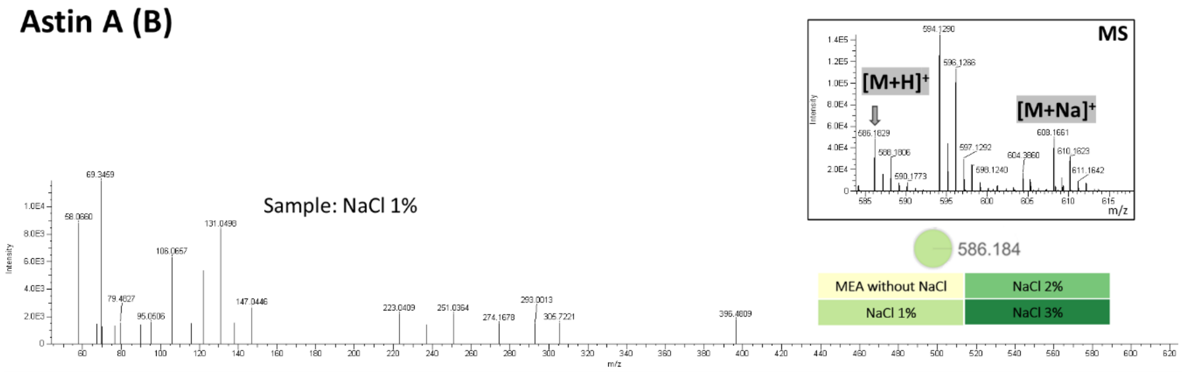

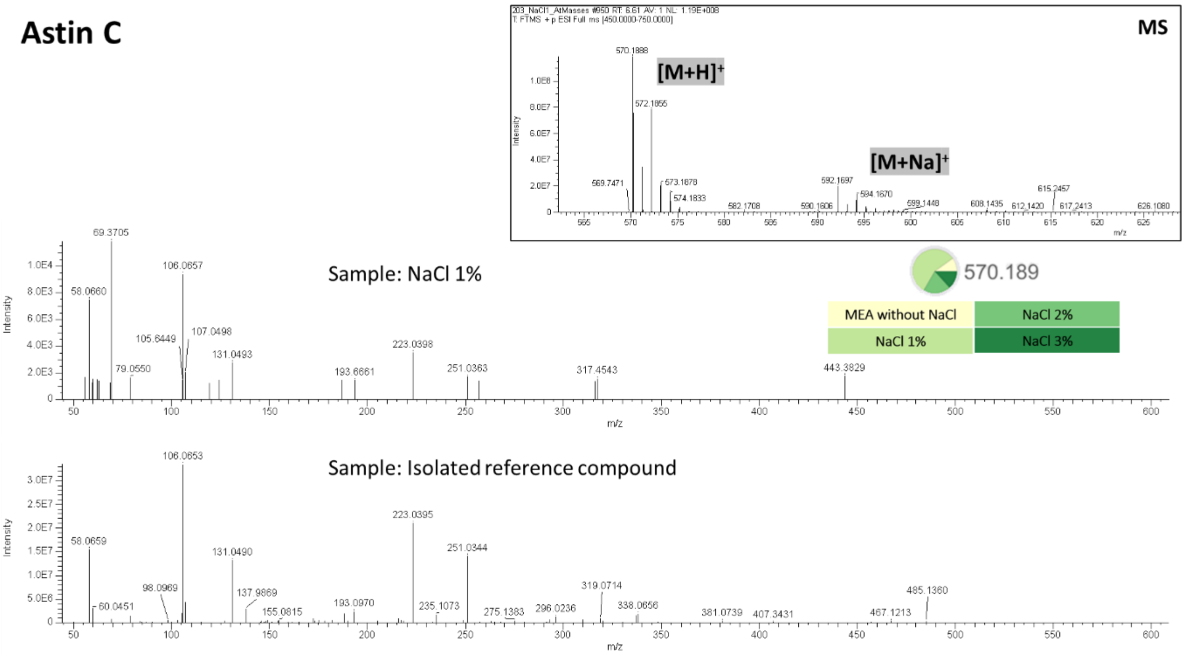

Astin F (M)

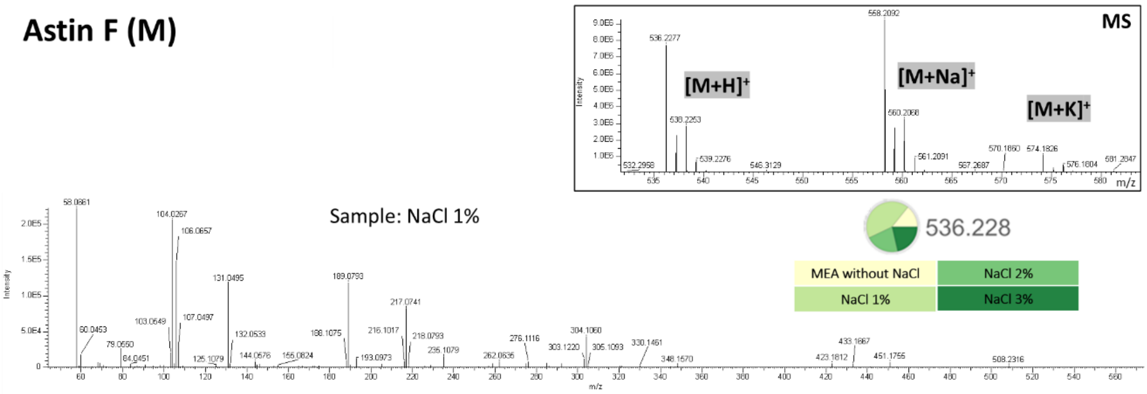

Astin G

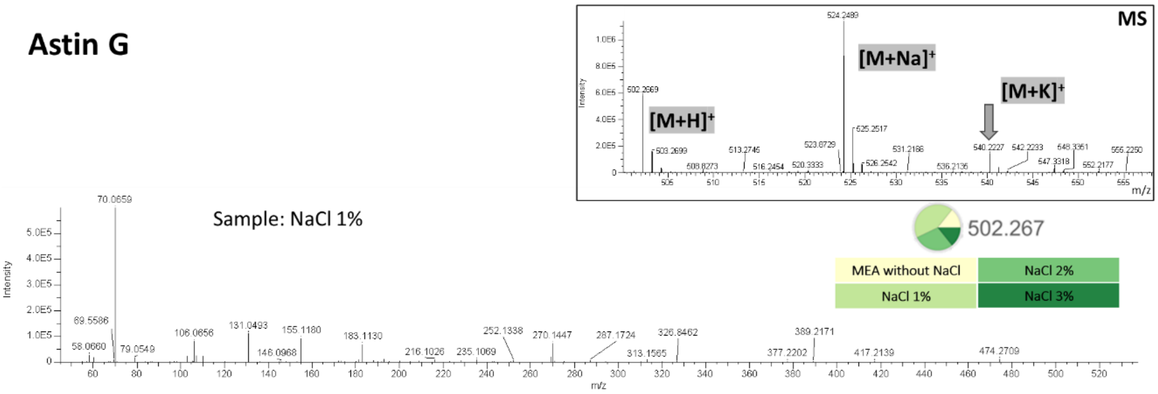

Astin I

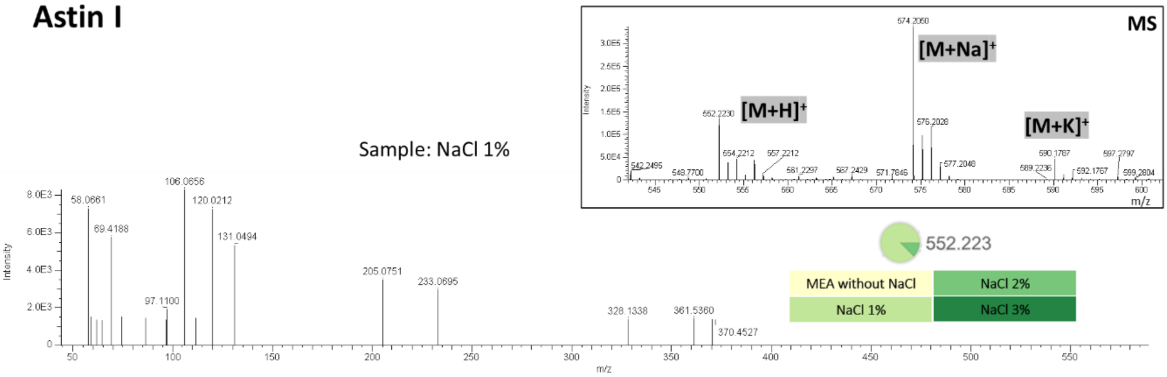

Astin R

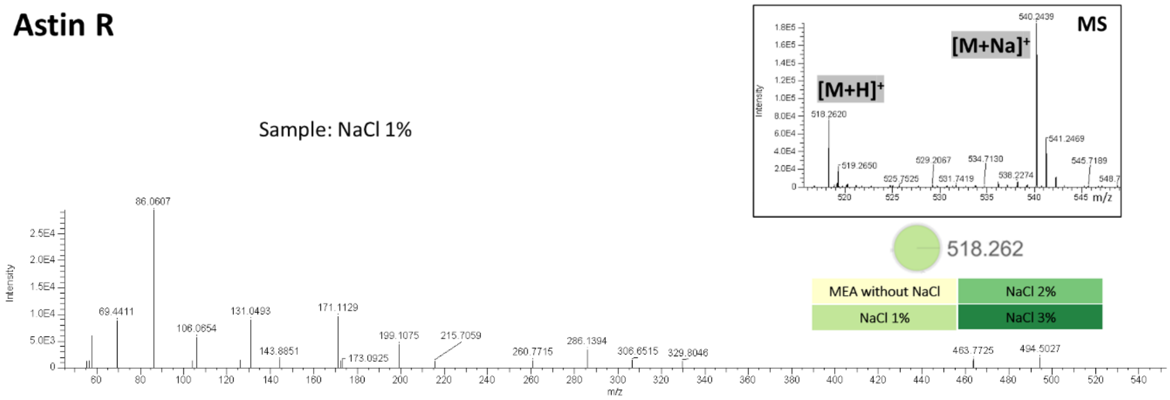

Astin S

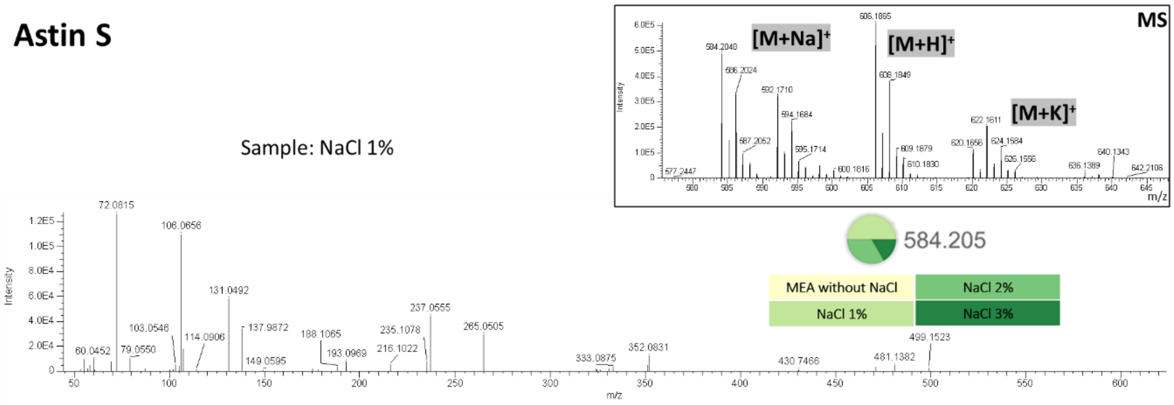

Asterinin D

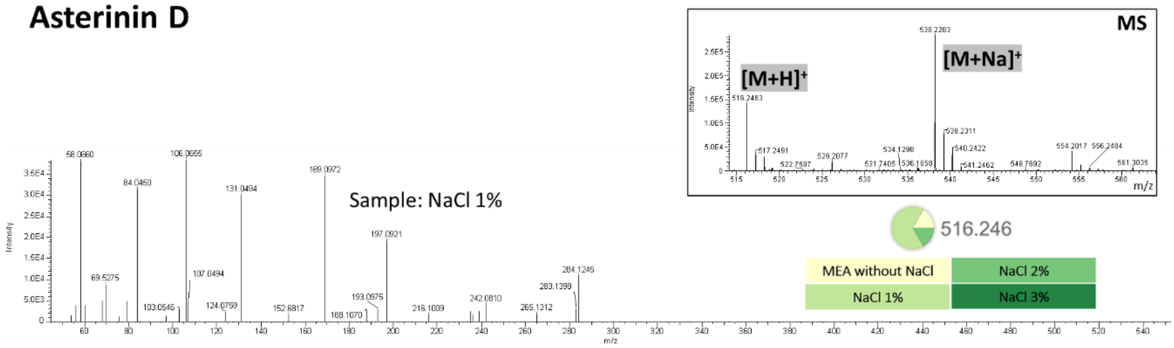

$C_{24}H_{33}Cl_2N_5O_5$

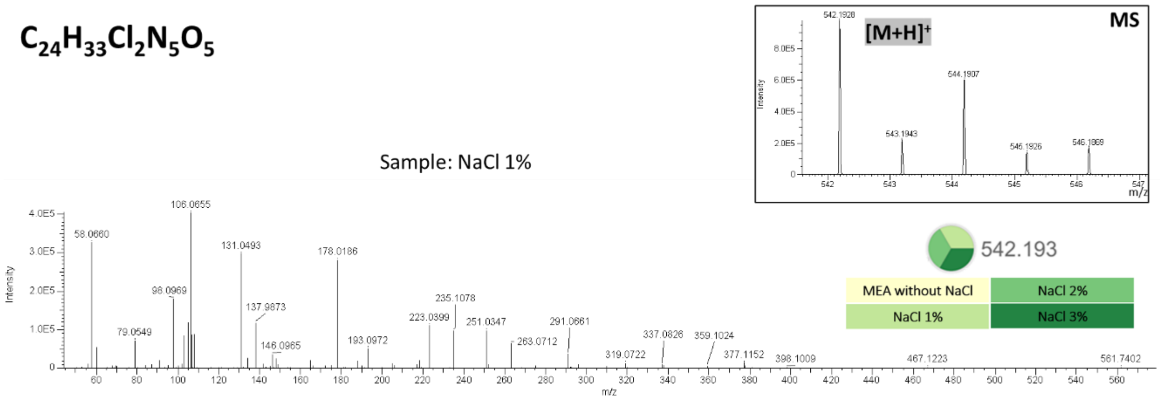

$C_{24}H_{31}Cl_2N_5O_6$

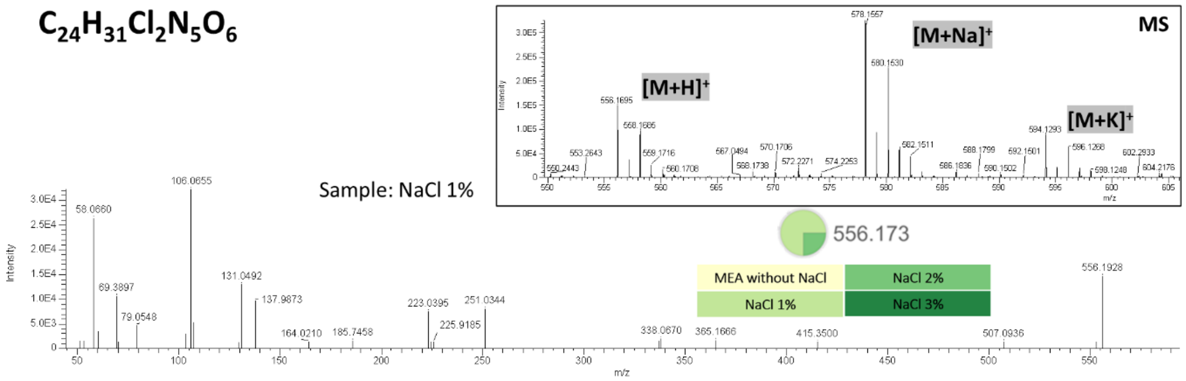

$C_{30}H_{37}ClN_5O_7$

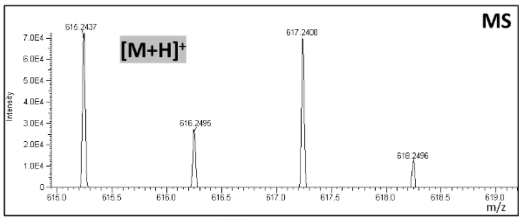

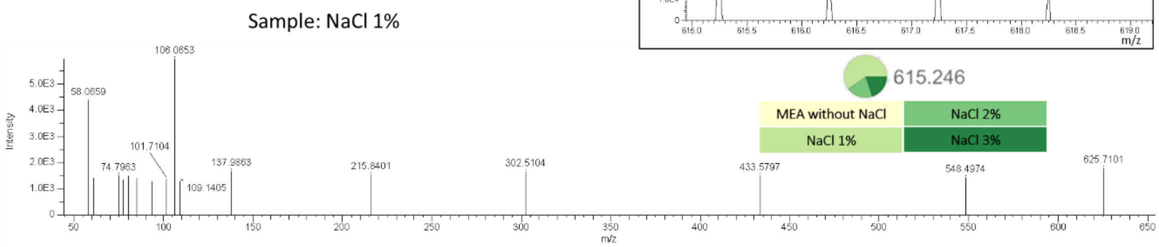

$C_{29}H_{37}Cl_2N_5O_9$

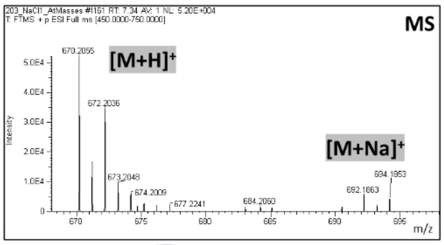

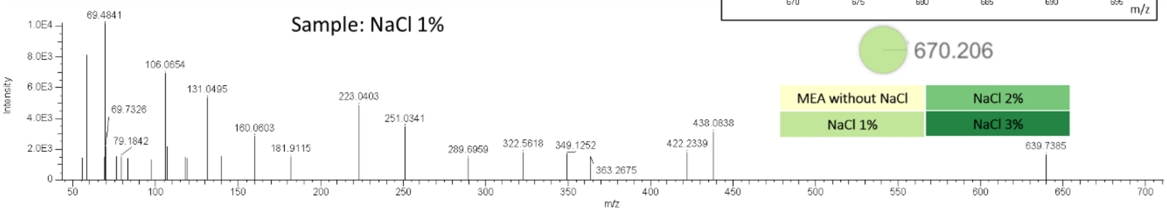

Not determined

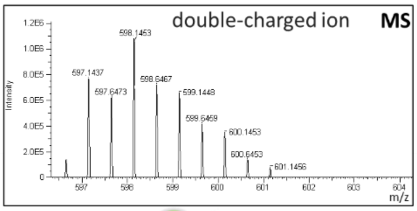

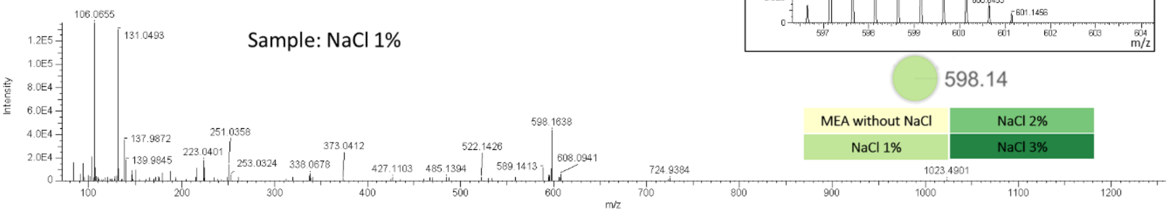

Figure S4. MS/MS data and main fragment interpretation of the postulated astins R and S.

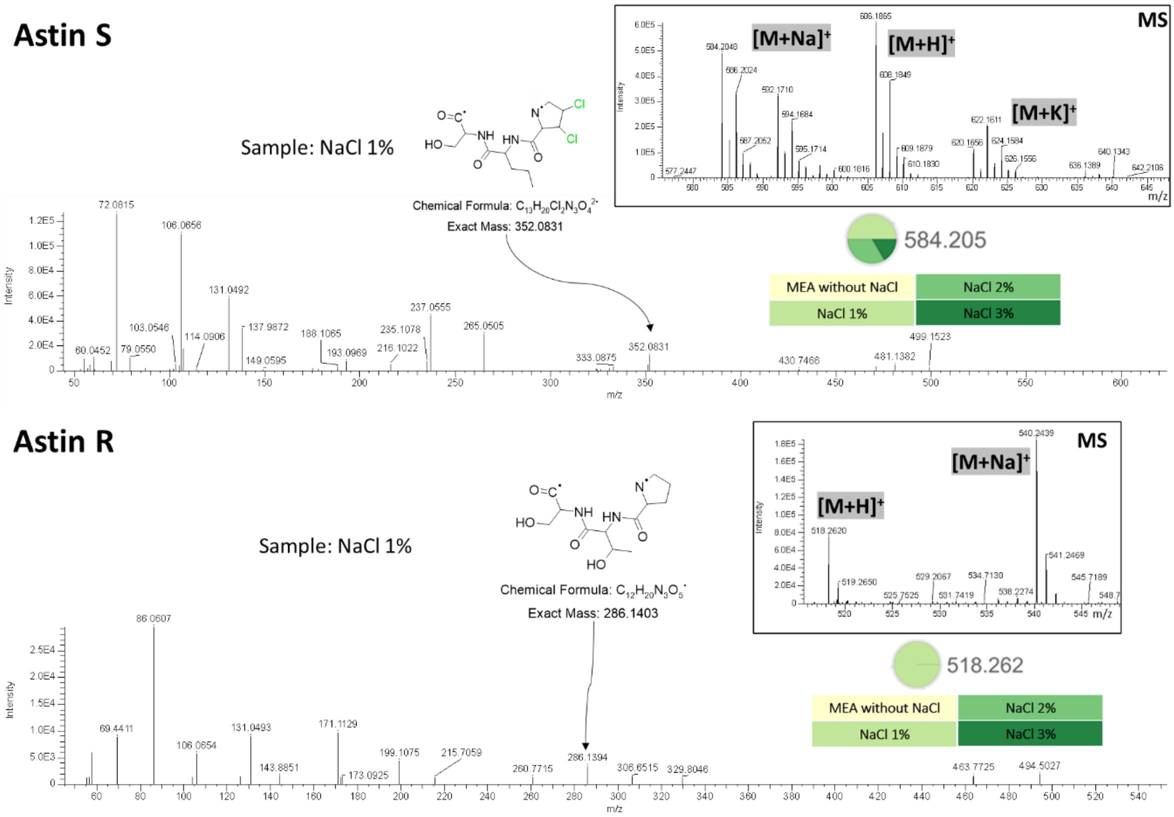

Supplementary text concerning the CARD-FISH (Fluorescence *in situ* hybridization with catalyzed reporter deposition) experiments

Materials and Methods

Sample preparation. For initial experiments to confirm that the developed probe is able to detect the fungus, an agar petri dish with *C. asteris* was superficially scraped to obtain mycelium and spores, which were placed on superfrost glass slides (VWR, Germany) for later FISH analysis. For the detection of *C. asteris* in plant sections, *A. tataricus* was collected in October 2022 and divided into different organs: flowers, leaves, stem, rhizome, and roots as described in the main manuscript. The collected tissues were embedded in 10% gelatin and stored in a freezer at -70 °C until sectioning. Tissues from *A. tataricus* were sectioned at -20 °C with a microtome (MICROM HM 500 M, from MICROM International GmbH, Germany), 2 µm thickness, and placed on superfrost glass slides. Subsequently, the samples were stored in a freezer at -70 °C until further use.

Fixation. A formaldehyde solution (4% in PBS) was used as a fixing solution. The slides were submerged in the solution at 4 °C for 1 h in the dark. Subsequently, the slides were washed with 1x PBS (pH 7.3) followed by EtOH 96% and allowed to air-dry. The samples were then stored at -20 °C until further processing.

Autofluorescence quenching and permeabilization. To reduce plant tissue autofluorescence, several treatments were applied; the respective solutions were prepared as follows: 10 mg/mL toluidine blue O in PBS, 10 mg/mL toluidine blue O in EtOH, 10 mg/mL $FeCl_3$ in PBS, 10 mg/mL $FeCl_3$ in EtOH, 10 mg/mL sodium borohydride in PBS, 0.25% ammonium in EtOH. These solutions were separately placed on top of the plant (toluidine blue O/PBS 1 min, $FeCl_3$/EtOH 1 min, borohydride/PBS 40 min, 0.25% ammonium/EtOH 1 h), then the slides were rinsed with PBS until the solution ran clear and subsequently washed with EtOH 96%. The tissues were embedded in low gelling point agarose 0.1% (Panreac AppliChem, ITW Reagents, Germany) and dried at 37 °C for 20 min. Thereafter, the samples were permeabilization with 100 µL of chitinase/PBS (1 mg/mL 1x PBS-SDS 1% pH 5.5, chitinase from Merck) for 15 min at 37% with a coverslip. Finally, they were rinsed with sterile distilled Water and allowed to air-dry. The treated tissues were photographed under a manual epifluorescence microscope (described above) with white light and fluorescence filters for TRITC (Ex 557 nm), DAPI (Ex 350 nm) and Alexa 488 (Ex 490 nm).

Inactivation of endogenous peroxidases. 100 µL H_2O_2 solution (3% in ultrapure water) was applied on top of fixed and permeabilized samples for 10 min at room temperature to quench the endogenous peroxidase activity of the plant, endophytic fungi or bacteria in samples. Subsequently, the slides were washed with 1x PBS for 5 min and distilled water for 1 min and allowed to air-dry.

Hybridization. The *in-situ* hybridization of *C. asteris* was performed using positive and negative oligonucleotide probes C_ast_HRP (5'-GATCCCCTAACTTTCCATCCTGAT-3') labeled with Alexa488 (00315807_1 DNA; *C. asteris* fungus SS rRNA-targeted) and NON_HRP (5'-ACTCCTACGGGAGGCAGC-3') (00315807_2 DNA), purchased from Biomers.net GmbH (Ulm, Germany). The hybridization buffer contained 900 mM NaCl, 20 mM Tris-HCl (pH 8.0), 10% dextran sulfate (wt/vol), 10, 20, or 30% (vol/vol) formamide (respectively), 10% blocking reagent and 0.02% sodium dodecyl sulfate (vol/vol).

The blocking reagent was prepared in maleic acid buffer (100 mM maleic acid, 150 mM NaCl, pH 7.5). 300 µL of hybridization buffer and 1 µL of probe working solution (8.5 pm.μL^{-1}) were added in a 0.5 mL reaction vial, and 100 µL of this solution were placed on top of the tissue, covered with a cover slip to improve the reaction and avoid drying. The reaction slides were incubated in hermetic chambers of 50 mL, saturated with a hybridization buffer, in an oven at 46 °C for 2 h. Then, the hybridized slides were transferred to a 50-mL chamber with prewarmed washing buffer and incubated at 48 °C for 15 min. The washing buffer was prepared with 22.5 mM NaCl, 5 mM EDTA (pH 8.0), 20 mM Tris-HCl (pH 8.0), and 0.01% SDS (wt/vol) (sodium dodecyl sulfate).

Tyramide signal amplification. After the buffer washing step, the hybridized slides were washed again with 1xPBS for 15 min at room temperature. Then, the slides were dabbed onto blotting paper tissue to remove excess buffer and covered with 100 µL of substrate mix, incubated at 46 °C for at least 45 min in a chamber within a MQW soaked paper in the dark. The substrate mix consisted of 1000 parts amplification buffer (0.1% 1xPBS [pH 7.3], 2M NaCl, 0.1% blocking reagent, Dextran Sulfate 10%, all in MQW), 10 parts freshly prepared 0.15% H_2O_2 in PBS, and 1 part Alexa 488 labeled tyramide (Jena Bioscience, Germany). The slides were washed with 1xPBS for 15 min at room temperature, followed by washing with ddH_2O for 3 min, and 96% ethanol for 1 min. Afterward, the slides were air dried and stained with 20 µL of DAPI mix (2 µg/mL DAPI (Carl Roth), 140 µL Vectashield (Biozol, Germany), 750 µL citiFluor (Electron Microscopy Science, US), 70 µL 1xPBS) and finally covered with a cover glass. The slides were stored at -20 °C and evaluated the next day as described in the methodology in the main manuscript.

Results

***C. asteris* detection by CARD-FISH.** Fluorescence *in-situ* hybridization (FISH) combined with catalyzed reporter deposition (CARD) is a well-known technique used to localize and identify microorganisms directly in their specific microenvironment. CARD has been demonstrated to increase the fluorescent label signal by more than 10-fold.[1] Using this technique, it has been possible to study interactions between plant pathogens directly on tissues, e.g. spores and hyphae from *Phytophthora cinnamomi* in apples and plant roots.[2] The technique involves 4 main steps: fixation/permeabilization, hybridization, washing, and visualization/detection,[3] where the first two steps must be optimized (Fig. S3, S4).

Optimization of the formamide concentration. One of the first parameters to optimize was the concentration of formamide, used to lower the melting point of the rRNA targets.[4] Although the concentration of formamide must be experimentally optimized for each probe, the mathFISH model was used to predict the formamide curve and the maximum hybridization efficiency for the designed probes.[5] The ideal concentration was predicted to be between 10 and 30% of formamide for our probe C_ast_HRP. As shown in Figure S5, the best result was obtained with a formamide concentration of 20%. In this experiment, the hyphae had a normal structure, and it was possible to distinguish spores and their nuclei from the dark background.

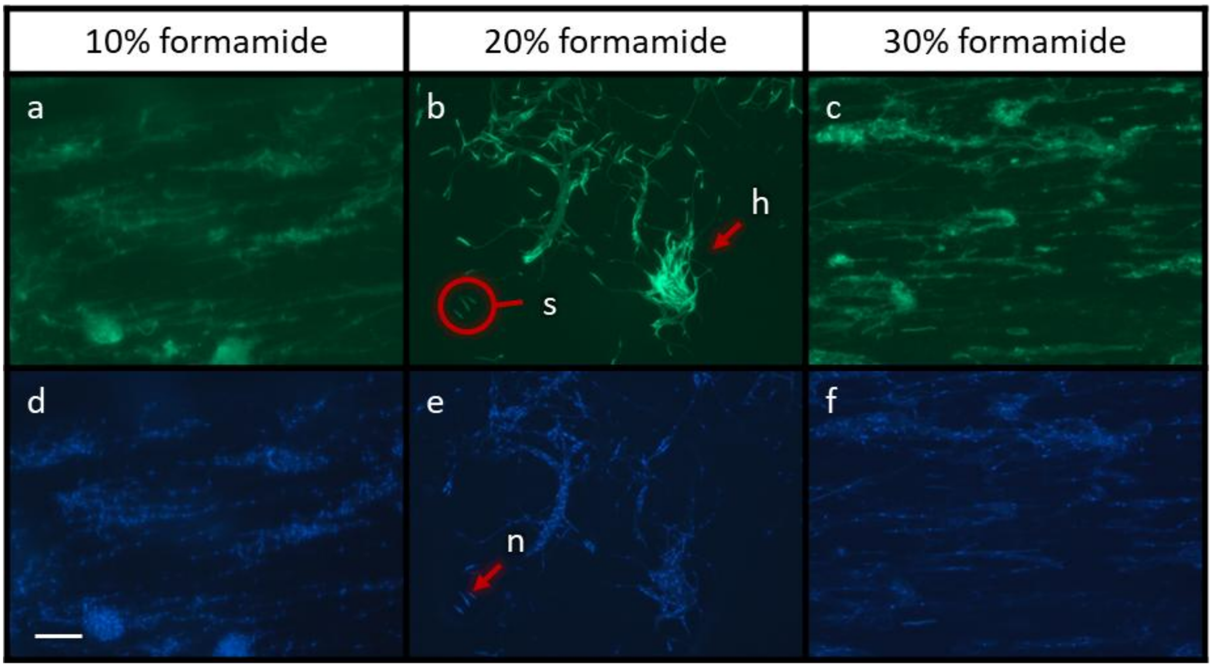

Figure S5. Micrographs of *C. asteris* after CARD-FISH hybridization (C_ast_HRP probe, labeled with AlexaFluor448, DAPI-stained) with different concentrations of formamide. a, d. 10% formamide. b, d. 20% formamide. c, f. 30% formamide. Displayed in channel green (3000 ms) and blue (120 ms). Hyphae (h), spores (s), and nuclei (n).

Optimization of permeabilization/fixation. Permeabilization/fixation is a key step in FISH experiments, but its optimization is difficult. This step should provide good probe penetration, preservation of the target RNA, maintenance of cell integrity and morphology.[6,7] For this purpose, formaldehyde (FA) was used in two different concentrations and two temperatures. In the experiments with 2% FA, at 4 °C and RT, cell degradation was always observed in the background (Figure S6a, c). In 4% FA at RT (Figure S6b), the green fluorescence was not uniform through the hyphae, hyphae segments were not effectively hybridized. Finally, the 4% FA concentration at 4 °C was found to be the best combination between temperature and FA concentration. The result indicated that under these conditions, the hyphae and the spores have uniform green fluorescence due to good hybridization and low background noise.

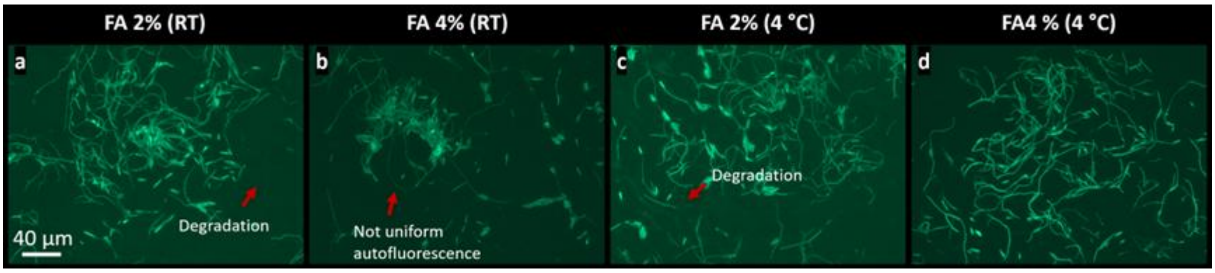

Figure S6. Micrographs of *C. asteris* after CARD-FISH hybridization (C_ast_HRP probe, labeled with AlexaFluor448) with different concentrations of formaldehyde and at different temperatures. Displayed in channel green (3000 ms).

Attempts to optimize autofluorescence quenching. One challenge when using fluorescence as a visualization method is the autofluorescence of tissues, especially when working with plant tissues. Autofluorescence originates from naturally present endogenous fluorescent compounds, e.g. coumarins, flavonoids, carotenoids, cellulose, chlorophyll (orange/red fluorescence), and lignin (blue/green fluorescence).[8] Especially the latter two are main constituents of plant cells and show high autofluorescence,[9,10] requiring dedicated FISH protocols that overcome this effect in plant tissue analysis.[11] Reducing the autofluorescence in plants is challenging due to the high chemical diversity of the compounds causing it. While some can be dissolved in organic solvents like methanol or hexane (depending on the polarity), lignin and cellulose, abundant in plant cell walls, are insoluble in most solvents.

For reducing the autofluorescence of the *A. tataricus* tissues, we evaluated different agents following two main strategies known in the field: modification of the fluorescent molecules by chemical reactions ($FeCl_3$, reacting with phenolic groups; borohydride, reducing aldehydes and keto-groups; ammonia-ethanol, inactivating pH-sensitive fluorochromes)[12] or quenching by staining biopolymers with toluidine blue O (TBO).[13,14] None of the treatments were able to completely abolish autofluorescence of the studied tissue (Figure S7). Our impression was that the best result was obtained with TBO in PBS, although the autofluorescence was still intense even after treatment. In the literature, this method has been used as a short-time staining procedure that produces autofluorescence quench.[2,13,15] To the best of our knowledge, it has not yet been used to prepare tissues for CARD-FISH experiments.

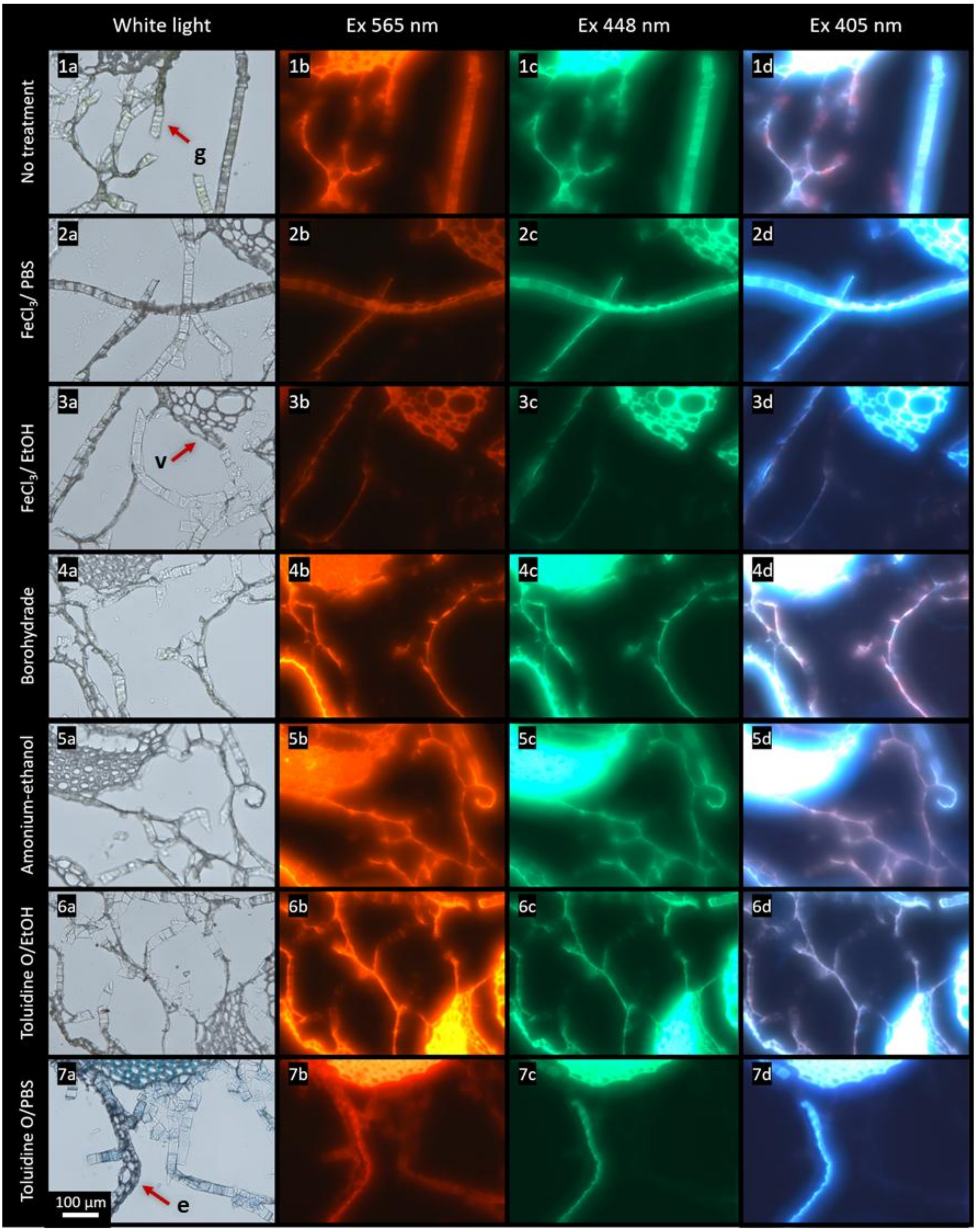

Figure S7. Micrographs of the transversal stem section from *A. tataricus* with treatments to reduce the autofluorescence on plant tissue (14 µm thickness). Tissues treated with agents to reduce autofluorescence. Epidermis (e), ground tissue system (g), and vascular tissue system (v). Displayed in brightfield and UV channels blue, green, and red.

Detection of *C. asteris* by CARD-FISH in plant tissue. Two probes were used for the CARD-FISH assay, one specifically targeting *C. asteris* and one as negative control. To allow comparison with the micrographs described in the main manuscript, a base rosette cross-section was used. In Figure S8 b and d, a comparison of the hybridized tissues with positive and negative probes revealed that the structures previously colored by LCB exhibit fluorescence upon hybridization with C_ast_HRP in the plant tissue. The same structures in the tissue hybridized with NON_HRP were less bright.

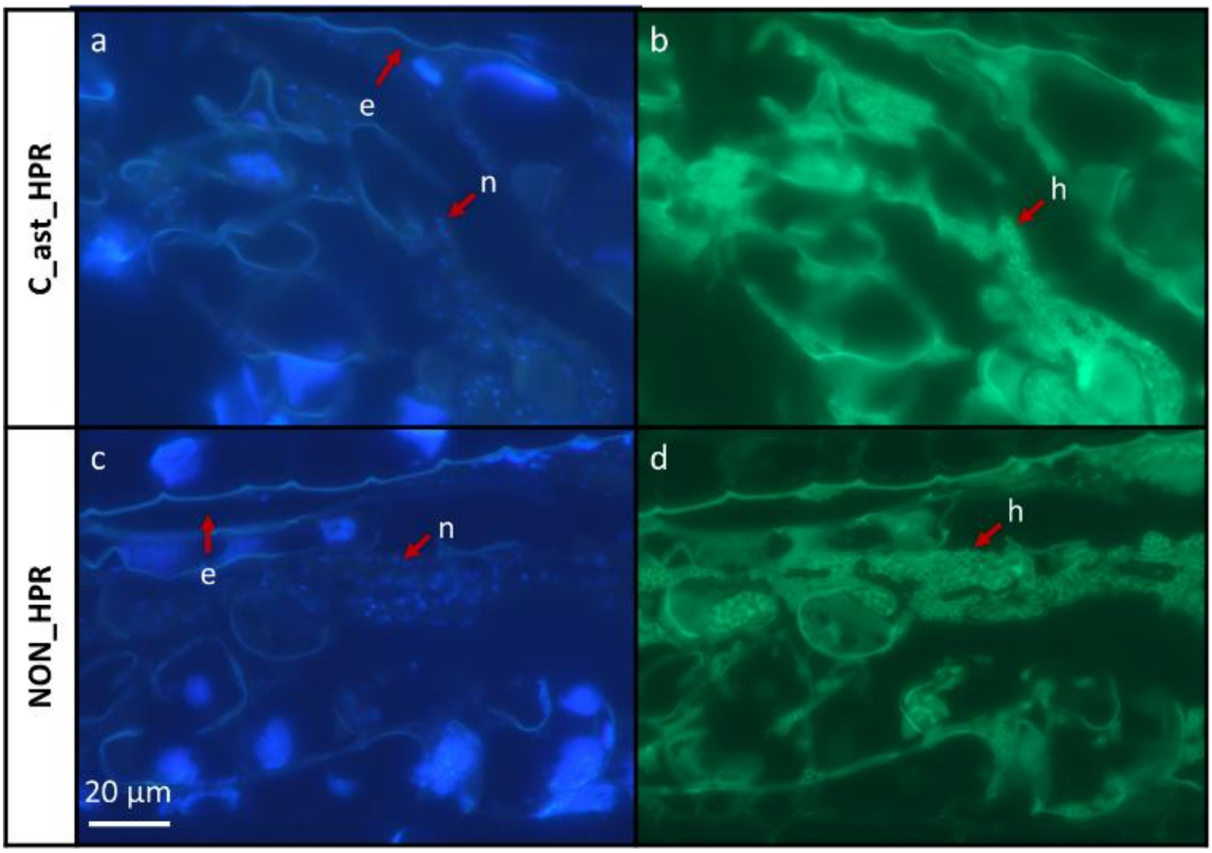

Figure S8. CARD-FISH visualization of *C. asteris* on *A. tataricus* tissue. a-c. hybridization with C_ast_HRP probe labeled with AlexaFluor448 and DAPI stain. d-f. hybridization with NON_HRP probe labeled with AlexaFluor448 and DAPI stain. Displayed in channels, blue (150 ms) and green (3000 ms). *A. tataricus* epidermis (e) and putative *C. asteris* hyphae (h) and nucleus (n).

The results were not conclusive. While the LCB-stained structures showed bright fluorescence when treated with the *C. asteris* probe, suggesting they were indeed *C. asteris*, they also showed fluorescence with the negative probe, probable due to autofluorescence that is also obvious from Figure S5. Thus, we could not unequivocally confirm that these structures were indeed the fungus *C. asteris*. These experiments would need further refinement and optimization.

Supplementary References

(1) Kubota, K. CARD-FISH for environmental microorganisms: technical advancement and future applications. *Microbes Environ* **2013**, *28* (1), 3–12. DOI: 10.1264/jsme2.me12107.

(2) Li, A. Y.; Crone, M.; Adams, P. J.; Fenwick, S. G.; Hardy, G. E. S. J.; Williams, N. The microscopic examination of *Phytophthora cinnamomi* in plant tissues using fluorescent *in situ* hybridization. *J Phytopathol* **2014**, *162* (11-12), 747–757. DOI: 10.1111/jph.12257.

(3) Amann, R.; Fuchs, B. M. Single-cell identification in microbial communities by improved fluorescence *in situ* hybridization techniques. *Nat Rev Microbiol* **2008**, *6* (5), 339–348. DOI: 10.1038/nrmicro1888.

(4) Fontenete, S.; Guimarães, N.; Wengel, J.; Azevedo, N. F. Prediction of melting temperatures in fluorescence in situ hybridization (FISH) procedures using thermodynamic models. *Crit. Rev. Biotechnol.* **2016**, *36* (3), 566–577. DOI: 10.3109/07388551.2014.993589.

(5) Yilmaz, L. S.; Parnerkar, S.; Noguera, D. R. mathFISH, a web tool that uses thermodynamics-based mathematical models for *in silico* evaluation of oligonucleotide probes for fluorescence *in situ* hybridization. *Appl Environ Microbiol* **2011**, *77* (3), 1118–1122. DOI: 10.1128/AEM.01733-10.

(6) Rocha, R.; Almeida, C.; Azevedo, N. F. Influence of the fixation/permeabilization step on peptide nucleic acid fluorescence *in situ* hybridization (PNA-FISH) for the detection of bacteria. *PloS one* **2018**, *13* (5), e0196522. DOI: 10.1371/journal.pone.0196522.

(7) Moter, A.; and Göbel, U. B. Fluorescence in situ hybridization (FISH) for direct visualization of microorganisms. *J. Microbiol. Methods* **2000**, *41* (2), 85–112. DOI: 10.1016/S0167-7012(00)00152-4.

(8) Croce, A. C. Light and autofluorescence, multitasking features in living organisms. *Photochem* **2021**, *1* (2), 67–125. DOI: 10.3390/photochem1020007.

(9) Donaldson, L. Autofluorescence in plants. *Molecules* **2020**, *25* (10). DOI: 10.3390/molecules25102393.

(10) Escamez, S.; Terryn, C.; Gandla, M. L.; Yassin, Z.; Scheepers, G.; Näsholm, T.; Sundman, O.; Jönsson, L. J.; Lundberg-Felten, J.; Tuominen, H.; Niittylä, T.; Paës, G. Fluorescence Lifetime Imaging as an In Situ and Label-Free Readout for the Chemical Composition of Lignin. *ACS Sustainable Chem. Eng.* **2021**, *9* (51), 17381–17392. DOI: 10.1021/acssuschemeng.1c06780.

(11) Peredo, E. L.; Simmons, S. Leaf-FISH: In situ hybridization method for visualizing bacterial taxa on plant surfaces. *Antibody-Drug Conjugates* **2021**, *2246*, 111–128. DOI: 10.1007/978-1-0716-1115-9_8.

(12) Werner Baschong; Rosmarie Suetterlin; and R. Hubert Laeng. Control of autofluorescence of archival formaldehyde-fixed, paraffin-embedded tissue in confocal laser scanning microscopy (CLSM). *J Histochem Cytochem* **2001**, *49* (12), 1565–1572. DOI: 10.1177/002215540104901210.

(13) Biggs, A. R. Detection of impervious tissue in tree bark with selective histochemistry and fluorescence microscopy. *Stain Technol.* **1985**, *60* (5), 299–304. DOI: 10.3109/10520298509113928.

(14) O'Brien, T. P.; Feder, N.; and McCully, M. E. Polychromatic staining of plant cell walls by toluidine blue O. *Protoplasma* **1964**, *59*, 368–373. DOI: 10.1007/BF01248568.

(15) Sakai, W. S. Simple method for differential staining of paraffin embedded plant material using toluidine blue O. *Stain Technol.* **1973**, *48* (5), 247–249. DOI: 10.3109/10520297309116632.
